# Recent Advances on *O*-Ethoxycarbonyl and *O*-Acyl Protected Cyanohydrins

**DOI:** 10.3390/molecules26154691

**Published:** 2021-08-03

**Authors:** Héctor Manuel Torres Domínguez, Luis Mauricio Hernández Villaverde, Ronan Le Lagadec

**Affiliations:** Instituto de Química, UNAM, Circuito Exterior s/n, Ciudad Universitaria, Coyoacán, Ciudad de México 04510, Mexico; ectttor@comunidad.unam.mx (H.M.T.D.); mauriciovillaverde@comunidad.unam.mx (L.M.H.V.)

**Keywords:** *O*-protected cyanohydrins, *O*-acyl cyanohydrins, *O*-ethoxycarbonyl cyanohydrins, cyanohydrin preparation, catalysis, ethylcyanocarbonates, carbocyanation of aldehydes

## Abstract

Ethoxycarbonyl cyanohydrins and *O*-acyl cyanohydrins are examples of *O*-protected cyanohydrins in which the protecting group presents an electrophilic center, contributing to additional reaction pathways. The first section of this review describes recent advances on the synthesis of *O*-ethoxycarbonyl and *O*-acyl protected cyanohydrins. Reactions using KCN or alkyl cyanoformates as the cyanide ion source are described, as well as organic and transition metal catalysis used in their preparation, including asymmetric cyanation. In a second part, transformations, and synthetic applications of *O*-ethoxycarbonyl/acyl cyanohydrins are presented. A variety of structures has been obtained starting from such protected cyanohydrins and, in particular, the synthesis of oxazoles, 1,4-diketones, 1,3-diketones, 2-vinyl-2-cyclopentenones through various methods are discussed.

                      **Contents**

**1.**  **Introduction**
**2.**  **Synthesis of *O*-Protected Cyanohydrins**
  2.1. Synthesis of Ethoxycarbonyl Cyanohydrins
  2.2. Synthesis of *O*-Acyl Cyanohydrins
  2.3. Synthesis of *O*-Aroyl Cyanohydrins
  2.4. Asymmetric Cyanation
    2.4.1. Synthesis of *O*-Acyl Cyanohydrins
    2.4.2. Synthesis of *O*-Methoxycarbonyl Cyanohydrins
    2.4.3. Synthesis of *O*-Ethoxycarbonyl Cyanohydrins
**3.**  **Synthetic Applications**
  3.1. Synthesis of Substituted Cyclohexenes and Cyclopentenes
  3.2. Synthesis of 4-Heteroaryloxazoles
  3.3. Synthesis of 2-Aminocyclopentanones and 2-Amino-4-azacyclopentanones
  3.4. Synthesis of Cinnamic Esters
  3.5. Synthesis of 4-Amino-2(5*H*)-furanones
  3.6. Synthesis of Substituted 2-Vinyl-2-cyclopentenones
  3.7. Synthesis of *O*-Acylcyanohydrins from *O*-(α-Bromoacyl)cyanohydrins
  3.8. Synthesis of Substituted Cyclopropylamines and 1,4-Diketones
  3.9. Synthesis of α,α-Disubstituted α-Amino-Acids
  3.10. Synthesis of 2-Hydroxy-2-Cyclopentenones
  3.11. Synthesis of Highly Functionalized Acyclic Ketones
  3.l2. Synthesis of Substituted 1,3-Diketones
  3.13. Synthesis of 2,4,5-Trisubstituted Oxazoles by Palladium Catalyzed C-H Activation
**4.**  **Conclusions**
**5.**  **Abbreviations**
**6.**  **References**

                      **List of Tables**

**Table 1.**  Cyanocarbonation of aldehydes
**Table 2.**  Cyanoethoxycarbonilation of aldehydes in ionic liquids
**Table 3.**  Cyanoethoxycarbonilation of aldehydes catalyzed by DMAP under solvent free conditions
**Table 4.**  Cyanation of aldehydes with ethyl cyanoformate catalyzed by DMAP
**Table 5.**  Cyanation of ketones with ethyl cyanoformate catalyzed by DMAP
**Table 6.**  One-pot synthesis of *O*-acetyl cyanohydrins from aldehydes via *O*-silylcyanohydrins in [bmim]BF_4_.
**Table 7.**  Synthesis of *O*-acyl cyanohydrins with TMSCN, acetic anhydride and aldehydes catalyzed by B(C_6_F_5_)_3_
**Table 8.**  Synthesis of cyanohydrin esters from aroyl chlorides
**Table 9.**  Asymmetric cyanosilylation of aldehydes catalyzed by a thiourea derivative and conversion to *O*-acetylcyanohydrins
**Table 10.**  Asymmetric acetylcyanation of aldehydes catalyzed by vanadium(V) complexes
**Table 11.**  Substrate scope of the asymmetric catalytic formation of cyanohydrin carbonates with complex **VII** in the presence of lutidine
**Table 12.**  Enantioselective cyanoformylation of aldehydes catalyzed by the Ti(O*^i^*Pr)_4_/**IX** system
**Table 13.**  Enantioselective cyanation of aldehydes catalyzed by alumminium complex
**Table 14.**  Investigation of the substrate scope of the carboxycyanation with pyrocarbonate and KCN
**Table 15.**  Synthesis of compounds **123**–**130** by addition of anions of ethyl carbonates of cyanohydrins to 2-cycloalkenones
**Table 16.**  Synthesis of aminofuranones via intramolecular Blaise reaction
**Table 17.**  Substrate scope for the cross-coupling of the *O*-(α-bromoacyl)cyanohydrin with boronic acid
**Table 18.**  Titanium-mediated addition of EtMgBr to nitriles
**Table 19.**  Addition of EtMgBr to acyl cyanohydrins
**Table 20.**  Addition of Grignard reagents to acylcyanohydrin
**Table 21.**  Two steps *versus* one step reaction to prepare 3-substituted-2-hydroxy-2-cyclopentenones
**Table 22.**  Scope of the rearrangement of *O*-aromatic acylated cyanohydrins
**Table 23.**  Rearrangements of *O*-aliphatic acylated cyanohydrins
**Table 24.**  Three components coupling reaction to form cyanohydrin derivatives

## 1. Introduction

**Scheme 1:**
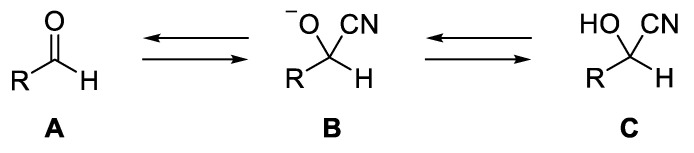
Reversible formation of cyanohydrin.

**Scheme 2:**
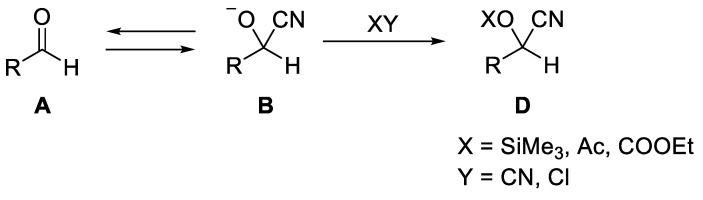
Irreversible formation of *O*-protected cyanohydrin.

Cyanohydrins and *O*-protected cyanohydrins are versatile building blocks in the preparation of important organic compounds including α-amino aldehydes, α-hydroxy acids, α-amino alcohols, and in the total synthesis of natural products and biologically active compounds [1,2]. A variety of methods for the asymmetric cyanation of aldehydes in the synthesis of cyanohydrins have been developed. Because of a reversible reaction in basic conditions, cyanohydrins are unstable, thus *O*-protected cyanohydrins are preferred (Scheme 1) [3]. In this case, intermediate **B** arising from the reversible addition of the cyanide ion to the aldehyde is trapped in an irreversible step to afford the *O*-protected cyanohydrin **D** (Scheme 2).

**Scheme 3:**
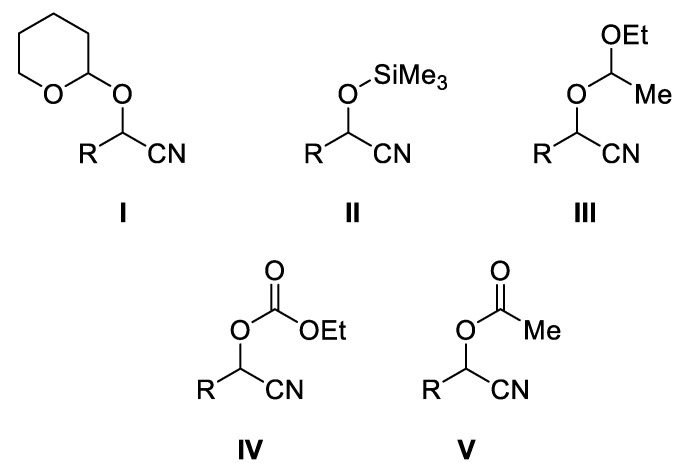
Examples of *O*-protected cyanohydrins.

Synthetic methodology to prepare cyanohydrins with protecting groups commonly used in organic chemistry, such as tetrahydropyranyl (**I**, THP) [4,5], trimethylsilyl (**II**, TMS) and 1-ethoxyethyl (**III**, EE) [6,7,8] have been described (Scheme 3). Such protected cyanohydrins can function as pronucleophiles in nucleophilic substitutions [9] and nucleophilic additions [10]. Ethoxycarbonyl cyanohydrins **IV** and *O*-acyl cyanohydrins **V** are examples of *O*-protected cyanohydrins in which the protecting group presents an electrophilic center. This structural characteristic imparts additional reaction pathways besides the observed in protected cyanohydrins with groups like TMS or THP. During the past years, reviews have discussed the preparation and synthetic applications of cyanohydrins [11,12,13,14,15,16,17,18]. In the reported studies, much effort has been brought on the asymmetric cyanation of aldehydes, either using transition-metal catalysts with chiral ligands or chiral organocatalysts. Additionally, the search for green alternatives of cyanide source has attracted much attention. In this review, we wish to focus on recent reports on the preparation and synthetic applications of *O*-protected cyanohydrins in which the protecting group is an alkoxycarbonyl or acyl moiety. Such derivatives present much interest due to the additional reactivity they impart to the cyanohydrin. The participation of these protected cyanohydrin in synthesis is also reviewed.

## 2. Synthesis of *O*-Protected Cyanohydrins

### 2.1. Synthesis of Ethoxycarbonyl Cyanohydrins

**Scheme 4:**
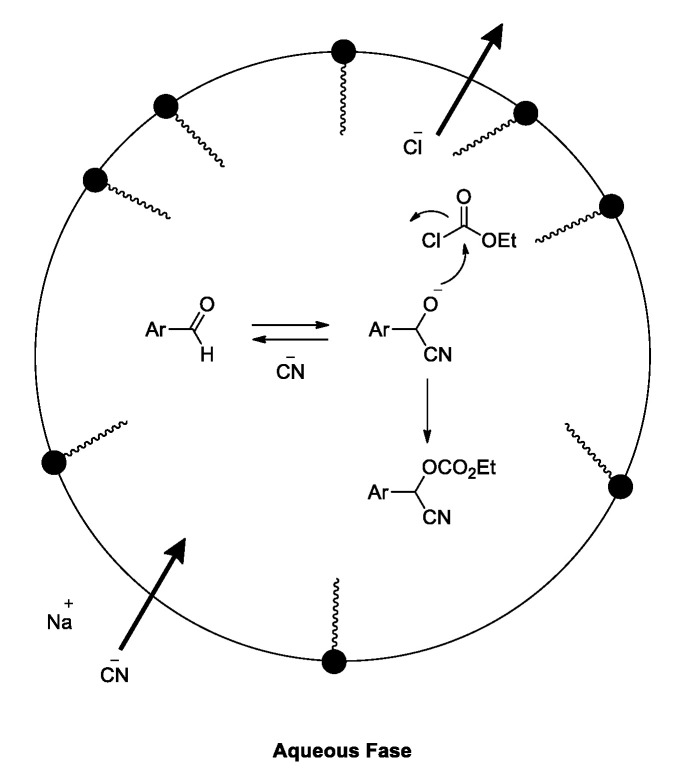
Proposed mechanism for the cyanocarbonylation of aldehydes in the presence of a surfactant in H_2_O.

Ethyl carbonates of cyanohydrins from aromatic aldehydes have been synthetized in water, using NaCN as the cyanide source and ethyl chloroformate as the ethylcarboxy group source. Surfactants are used to facilitate the incorporation of the organic reagents into the aqueous media, as shown in Scheme 4. After screening studies with 4-methylbenzaldehyde, dodecyltrimethyl ammonium chloride (DTMAC) has been chosen as the most efficient surfactant. Table 1 summarizes the scope of the method. High yields of products are obtained for electron-withdrawing groups (entries 2, 3, 7, 8, 12) and electron-donating groups (entries 4–6, 9–11) in substituted benzaldehydes. Heterocyclic benzaldehydes (entries 13, 14) also give high yields. Easy scale up of the reaction to multigrams, short reaction times, mild reaction conditions and facile isolation of the products characterized this synthetic methodology [19].

*N*-methyl-*N*′-alkyl imidazolium salts as ionic liquids have been employed in the cyanoethoxycarbonylation of aldehydes [20]. Optimization studies of the reaction between benzaldehyde and ethyl cyanoformate in the presence of an imidazolium salt as a catalyst at room temperature showed that C-5 alkyl chain length with Br^−^ ion as counterion in solvent-free conditions can produce high yields of *O*-ethoxycarbonyl mandelonitrile. Table 2 shows the scope of the method. Both electron-donating groups (entries 2–6) and electron-withdrawing groups (entries 7–9) in substituted benzaldehydes give excellent yields. Steric hindrance (entries 2 and 5) appears not to influence the reaction. With *p-tert*butylbenzaldehyde, the yield lowers to 91% (entry 11), while α, β-unsaturated (entry 10) and aliphatic aldehydes (entries 12 and 13) also produce yields higher than 90%.

**Scheme 5:**
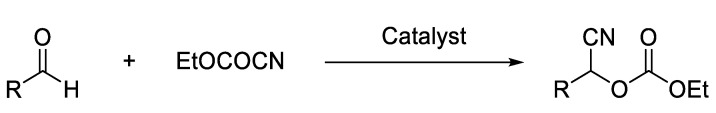
Synthesis of cyanohydrin carbonates from ethyl cyanoformate [21].

**Scheme 6:**
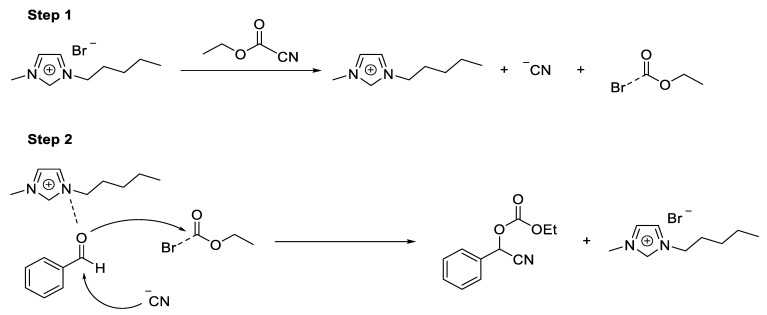
Proposed mechanism for the cyanoethoxylation of benzaldehyde in ionic liquids [21].

Kinetic and mechanistic studies in the formation of ethyl carbonates of cyanohydrins by ethyl cyanoformate addition to aldehydes catalyzed by amines have established the mechanism of the reaction (Scheme 5) [21]. A ^13^C NMR study of the reaction shows that the Br^−^ counterion interacts with ethyl cyanoformate to form the acylbromide and generates CN^−^, which adds to the carbonyl activated by the imidazolium cation (Scheme 6). When PF_6_^−^ counterion is used the reaction does not take place. Additional experiments showed the reusability of the catalyst up to eight catalytic runs without depletion of the yield of the reaction.

**Scheme 7:**
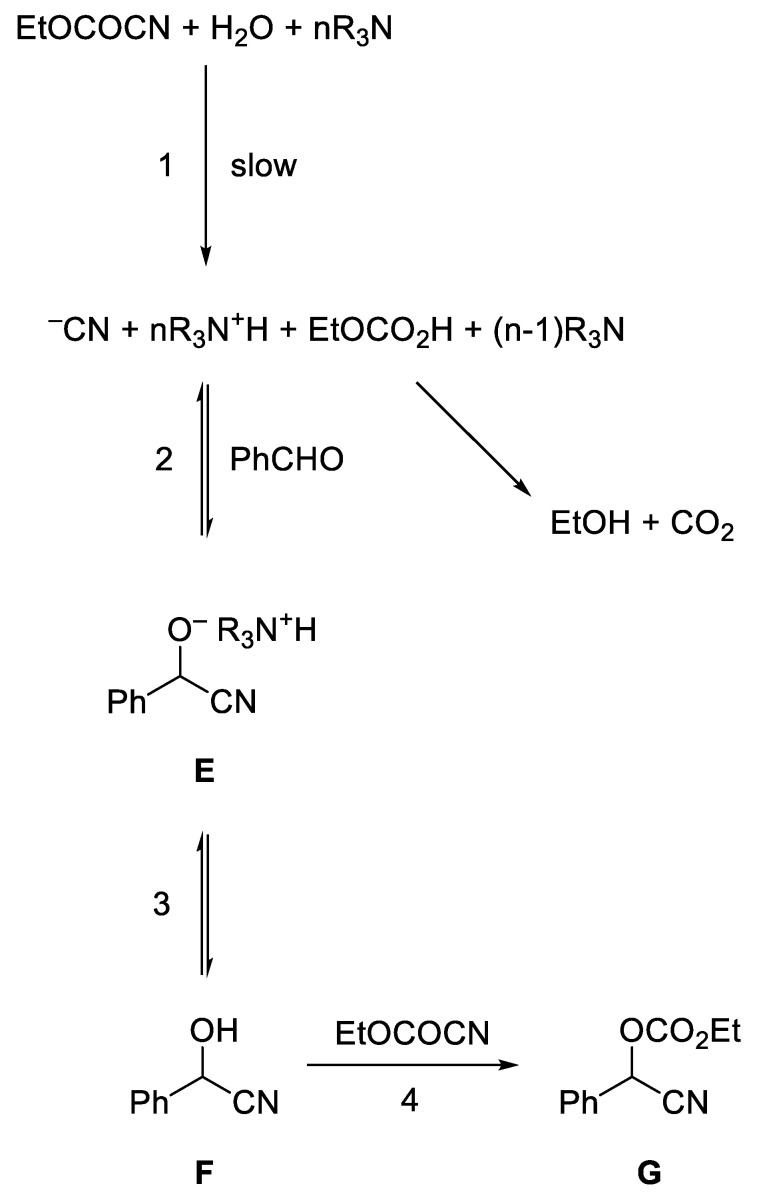
Proposed mechanism for the synthesis of cyanohydrin carbonates catalyzed by amines [21].

It has been determined that the reaction between benzaldehyde and ethyl cyanoformate catalyzed by amines follows the mechanism shown in Scheme 7 [21]. The first step is the irreversible slow hydrolysis of ethyl cyanoformate by adventitious water to generate the cyanide ion. The tertiary amines in this step serve as a Brønsted base, forming hydrogen bonding with a molecule of water. Kinetic studies confirm this step as rate determining in which no aldehyde is involved. The following step is the reversible nucleophilic addition of the cyanide ion to benzaldehyde to produce the cyanohydrin alkoxide **E**, which, by a reversible protonation from the ammonium ion, generates the cyanohydrin **F**. Finally, in an irreversible step, the cyanohydrin carbonate **G** is formed by reaction of **F** with ethyl cyanoformate. The established dependence between the catalytic activity of the amines and their pK_a_H is in accordance with this mechanism. This fact implies that amines like amidines or guanines with higher pK_a_H would be more convenient.

DMPA was used as an organic catalyst for the cyanoethoxycarbonylation of aromatic and aliphatic aldehydes to produce, in solvent-free conditions at room temperature, high yields of *O*-ethoxycarbonyl cyanohydrins [22]. This protocol shows advantages as simple reaction conditions, easy product isolation and environmentally friendly. The scope of the method is shown in Table 3.

**Scheme 8:**
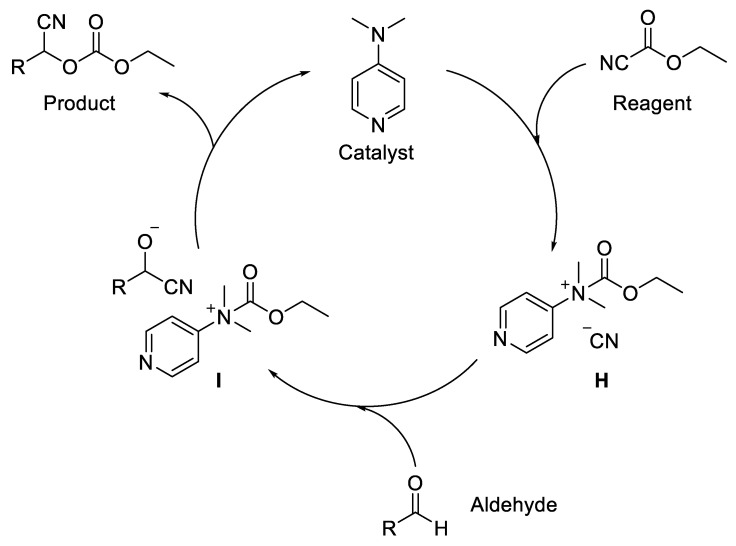
Proposed mechanism for the cyanoethoxycarbonylation of aldehydes catalyzed by DMAP [22].

When pyridine or 2,6-dimethylpyridine were used as catalyst instead of DMAP, the reaction does not proceed with benzaldehyde and ethylcyanoformate, while with triethylamine the product was obtained in 92% yield after 120 min. These results imply the intervention of a tertiary amine as a catalyst. The mechanism involves the formation of complex **H** from the reaction of DMAP with ethyl cyanoformate, followed by addition of CN^−^ to the aldehyde to give compound **I** which decomposes into the product and regenerates DMAP (Scheme 8).

With lower concentrations of DMAP and acetonitrile as solvent, *O*-ethoxycarbonyl cyanohydrins have been synthetized from aldehydes and ketones [23]. Table 4 shows the results. With aliphatic aldehydes (entries 1–3), a concentration of 1 mol % of DMAP was used to obtain good to high yields of ethyl carbonates of cyanohydrins. Aromatic aldehydes (entries 4–10) are less reactive and require a concentration of 5 mol % of DMAP.

With ketones, in absence of solvent, a DMAP concentration of 10 mol % was necessary to obtain good yields of the protected cyanohydrins (Table 5). When the reaction was carried out using cyclopentanone in acetonitrile (entry 1) the yield of the product was only 20%. Steric effects (entry 6) are present and lower the yield, and low reactive acetophenone gives only 30% of product (entry 4). Pyridine and *N*,*N*-dimethylaniline were unreactive under these conditions.

### 2.2. Synthesis of O-Acyl Cyanohydrins

**Scheme 9:**
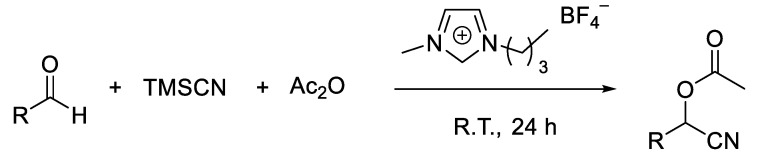
One-pot synthesis of *O*-acetyl cyanohydrin in ionic liquid [24].

*O*-acetyl cyanohydrins are synthetized from aldehydes in one step via the formation of *O*-silyl cyanohydrins in the presence of acetic anhydride and ionic liquids. No activator or Lewis catalyst is required as shown in Scheme 9. By screening various imidalozium based ionic liquids with benzaldehyde, [bmin]BF_4_ was found to give the highest yields [24].

Table 6 summarizes the results with a range of aldehydes. In general, good to high yields of products are obtained under mild reaction conditions, only *p*-cyanobenzaldehyde (entry 5) and 2-thiophenecarbaldehyde (entry 9) give yields lower than 80%. 

When tris(pentafluorophenyl)borane is used as a catalyst for the same one-pot three components coupling reaction to afford *O*-acetyl cyanohydrin from aldehydes or ketones and trimethylsilyl cyanide in solvent-free conditions at room temperature, lower yields are obtained. The scope of the reaction is shown in Table 7. When Bz_2_O is used instead of Ac_2_O, a lower yield is obtained (entry 2). Two electron-donating groups favors the reaction (entry 6). With *p*-nitrobenzaldehyde (entry 8) a longer reaction time is required, and the product is obtained in only 71% yield [25].

### 2.3. Synthesis of O-Aroyl Cyanohydrins

Aroyl chlorides can serve as starting materials in the synthesis of *O*-aroyl cyanohydrins using potassium cyanoferrate(II) as a cyanide source in the presence of a promoter of the reaction [26]. Several Lewis nucleophiles were tested as promoters. When pyridine and triethylamine were used, no reaction was observed, with triphenylphosphine and tributylphosphines in THF yields of **40** and 88% of the cyanohydrin esters were obtained respectively. Table 8 summarizes the scope of this method. Aroyl chlorides bearing electron-withdrawing substituents (entries 2–6) afford yields higher than 80%, while aroyl chlorides with electron-donating substituents (entries 7–9) give slighter lower yields. With 2-furoyl chloride (entry 10) the corresponding cyanohydrin ester was obtained in 78% yield.

**Scheme 10:**
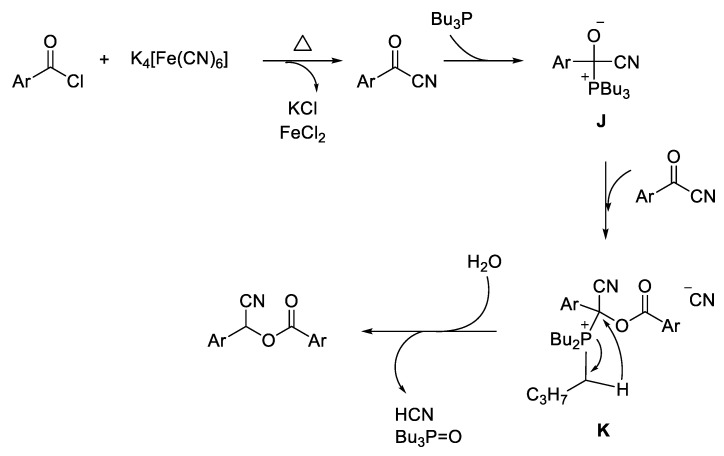
Proposed mechanism for the synthesis of cyanohydrin esters from aroyl chlorides [26].

The proposed mechanism (Scheme 10) involves various steps in one pot. First, the formation of the aroylcyanide from the reaction between 0.5 mol of K_4_[Fe(CN)_6_] and the aroyl chloride. In a second reaction, the aroyl cyanide thus formed reacts with the tributylphosphine to produce intermediate **J**, whichreacts with a second molecule of aroylcyanide to form **K**. Intramolecular donation of a hydride from one butyl group of the phosphine to the C bonded to the CN group affords, after hydrolysis from the atmosphere moisture, the cyanohydrin ester.

### 2.4. Asymmetric Cyanation

#### 2.4.1. Synthesis of *O*-Acyl Cyanohydrins

**Scheme 11:**
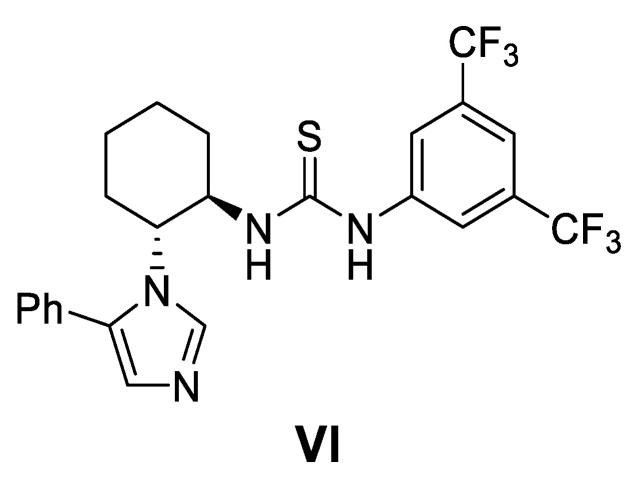
Structure of thiourea catalyst **VI** [27].

Asymmetric synthesis of *O*-acetyl cyanohydrins has been developed by a cooperative thiourea- Brønsted acid catalytic system [27]. Screening studies revealed that thiourea derivative **VI** (Scheme 11), with benzoic acid was the optimum selection. NMR and computational studies revealed that the function of the benzoic acid is to fix, via hydrogen bonding, the conformation of flexible thiourea by forming a bifunctional thiourea/benzoic acid complex. The asymmetric step involves the formation of the *O*-silyl cyanohydrin which is hydrolyzed with HCl and acetylated by Ac_2_O. Moderate to good yields of asymmetric cyanohydrins are obtained with moderate to low *ee* (Table 9). 

#### 2.4.2. Synthesis of *O*-Methoxycarbonyl Cyanohydrins

**Scheme 12:**
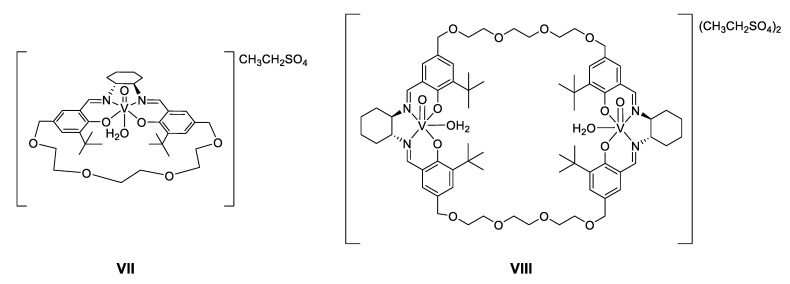
Structure of macrocyclic vanadium(V) catalysts **VII** and **VIII** [27].

Chiral protected cyanohydrins have also been obtained by the use of transition-metal complexes as catalysts in the asymmetric cyanation of aldehydes [28]. Chiral macrocyclic V(V)-salen complexes **VII** and **VIII** have been used as catalysts with KCN/NaCN and aldehydes in the preparation of chiral *O*-ethoxycarbonyl and *O*-acetyl protected cyanohydrins (Scheme 12). From studies with mononuclear Ti-salen complexes acting as bimetallic species, it was envisioned that in bimetallic V(V)-salen complexes, one V can activate the cyano group and a second V activates the aldehyde. Complex **VIII** exhibits two salen units linked by a polyether chain in which the crown ether-like chains function as trapping centers for K^+^/Na^+^ ions and activating KCN/NaCN.

Complexes **VII** and **VIII** catalyze the asymmetric cyanation of both aromatic and aliphatic aldehydes in the presence of KCN and acetic anhydride. Table 10 summarizes the scope of this method. In general, substituted aromatic aldehydes with both electron-donating and withdrawing groups gave the *O*-acetyl cyanohydrin with good to excellent *ee*.

#### 2.4.3. Synthesis of *O*-Ethoxycarbonyl Cyanohydrins

Subsequent application of the method with catalyst **VII** for the ethyl cyanoformylation of aldehydes uses 2,6-lutidine as a co-catalyst. A variety of aromatic and aliphatic aldehydes afford the desired products in good to excellent yields and *ee* higher than 85%. Table 11 summarizes the results.

**Scheme 13:**
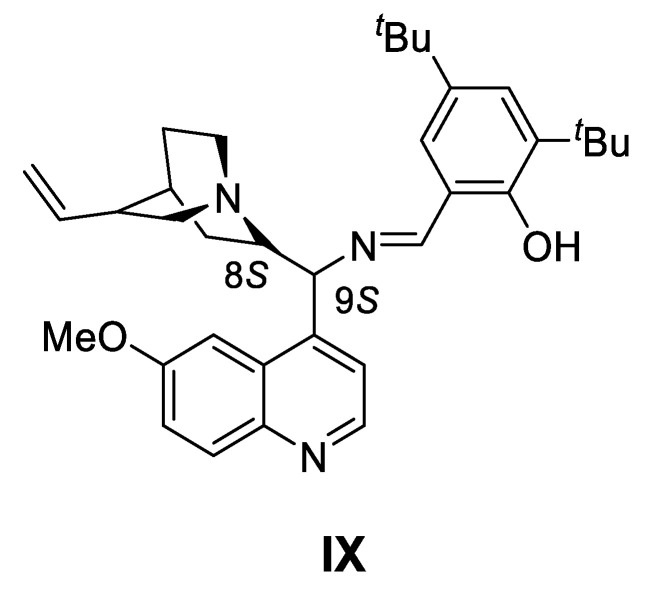
Structure of a Schiff base ligand used with Ti(O*^i^*Pr)_4_ [29].

A bifunctional Ti/Schiff base ligands from cinchona alkaloids and salicylaldehyde derivatives have been developed as catalysts in the enantioselective cyanoformylation of aldehydes with NCCOOEt (Scheme 13) [29]. After screening the effect of the ligands, solvent, concentration of the aldehyde and reaction temperature, the optimized reaction conditions were established. With ligand **IX** and Ti(O*^i^*Pr)_4_, the reaction proceeded with high yields and good enantioselectivity. Table 12 shows the scope of the reaction. Both electron-donating and electron-withdrawing substituted benzaldehydes give high yields of product with good enantiomeric excesses.

**Scheme 14:**
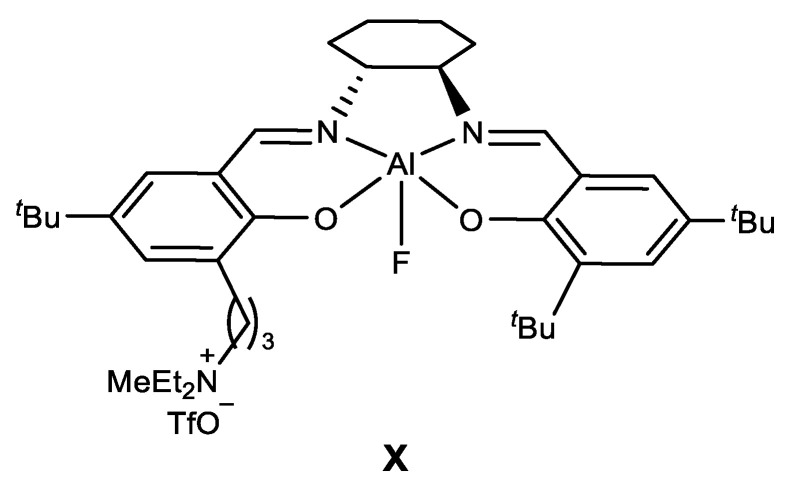
Structure of [Al-F]OTf catalyst **X** [30].

Al-F-salen complex **X** (Scheme 14) has been developed to increase the reactivity and enantioselectivity of the cyanation of aldehydes with ethyl cyanoformate to form *O*-ethoxycarbonylcyanohydrins. The incorporation of an aprotic onium moiety (ammonium ion) to the complex in addition to the Al-F Lewis acidic center converts **X** in a bifunctional cooperative catalyst. With these characteristics of the catalyst, the cyanation reaction is facilitated by a nucleophilic attack of a loosely bounded cyanide anion with the ammonium center to the carbonyl of the aldehyde activated by the Al center [30].

Table 13 summarizes the results with various aldehydes. A catalytic amount of KCN is necessary for the reaction to take place. Electron-donating and electron-withdrawing substituted benzaldehydes produce the cyanohydrins with high yields (entries 2–7 and 14–18). With 4-*^t^*Bu groups a moderate yield is obtained (entry 8). Cinnamaldehydes (entries 20 and 21) react with high yields, as well as aliphatic aldehydes (entries 24–28). The method presents high enantioselectivities and TONs of up to 10^4^.

When KCN is used as the only cyanide source with ethyl pyrocarbonate, rather similar results are obtained (Table 14). The method tolerates both electron-donating (entries 4–11) and electron-withdrawing substituted aldehydes (entries 12–14). With aliphatic aldehydes (entries 22 and 23) the enantioselectivity of the reaction is moderate.

## 3. Synthetic Applications

### 3.1. Synthesis of Substituted Cyclohexenes and Cyclopentenes

**Scheme 15:**
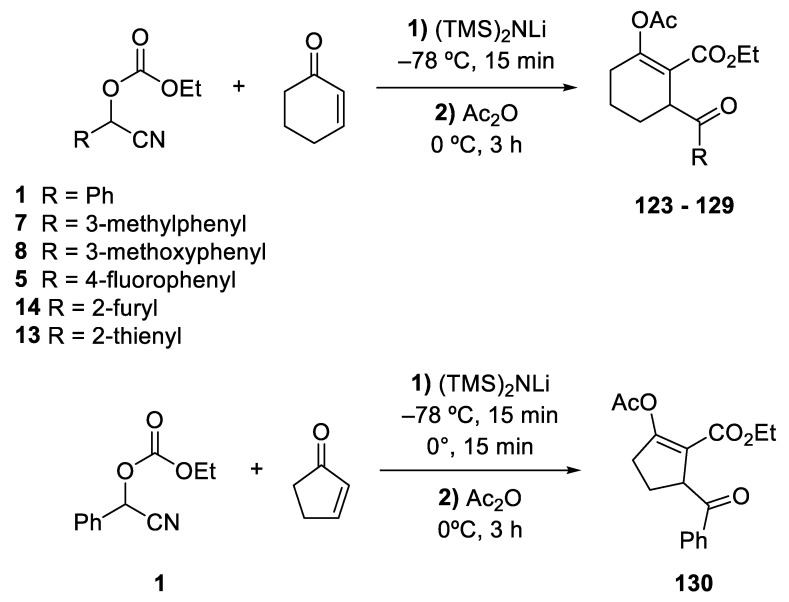
One step synthesis of cyclohexenes **123**–**129** and cyclopentene **130** from cyanohydrin carbonates [31].

Ethyl carbonates of cyanohydrins function as pronucleophiles with an additional electrophilic center located at the carbonyl group of the carbonate ester. These characteristics make them capable to participate in multiple steps reactions like domino reactions [31]. For instance, a one-pot Michael addition of anions of ethyl carbonates of cyanohydrins to conjugated 2-cycloalkenones followed by an intramolecular Claisen-type condensation have been reported (Scheme 15). Table 15 summarizes the scope of the reaction. Ethyl carbonates of cyanohydrins from aromatic aldehydes are obtained in overall good yields. Carbonates of cyanohydrins from benzaldehyde and substituted benzaldehydes (entries 1–4) give yields ≥ 69% and carbonates of cyanohydrins from heterocyclic aldehydes (entries 5 and 6) give lower yields when reacting with 2-cyclohexenone. The reaction is sensitive to the steric nature of the cycloalkenone, thus 4,4-dimethyl-2-cyclohexenone does not react under such conditions. When 2-cyclopentenone was used (entry 8) the corresponding substituted cylopentene **130** was prepared in good yield.

**Scheme 16:**
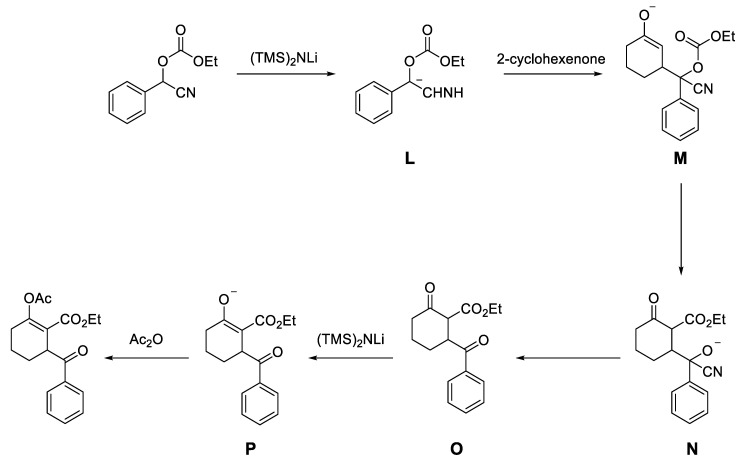
Proposed mechanism for the Michael addition-Claisen-type condensation of anions of ethylcarbonate of mandelonitrile to 2-cyclohexenone [31].

Scheme 16 illustrates a plausible mechanism for the reaction of ethylcarbonate of mandelonitrile **1** and 2-cyclohexenone. The first step involves the formation of the anion **L** of the carbonate of cyanohydrin, which react through a Michael addition with 2-cyclohexenone to produce **M**. An intramolecular attack of the enolate in **M** to the carbonyl group generates **N** which, after elimination of ^−^CN, forms the cycloalkanone **O**. A second equivalent of (TMS)_2_NLi produces the enolate **P** which is finally trapped with acetic anhydride to give the desired product.

### 3.2. Synthesis of 4-Heteroaryloxazoles

**Scheme 17:**
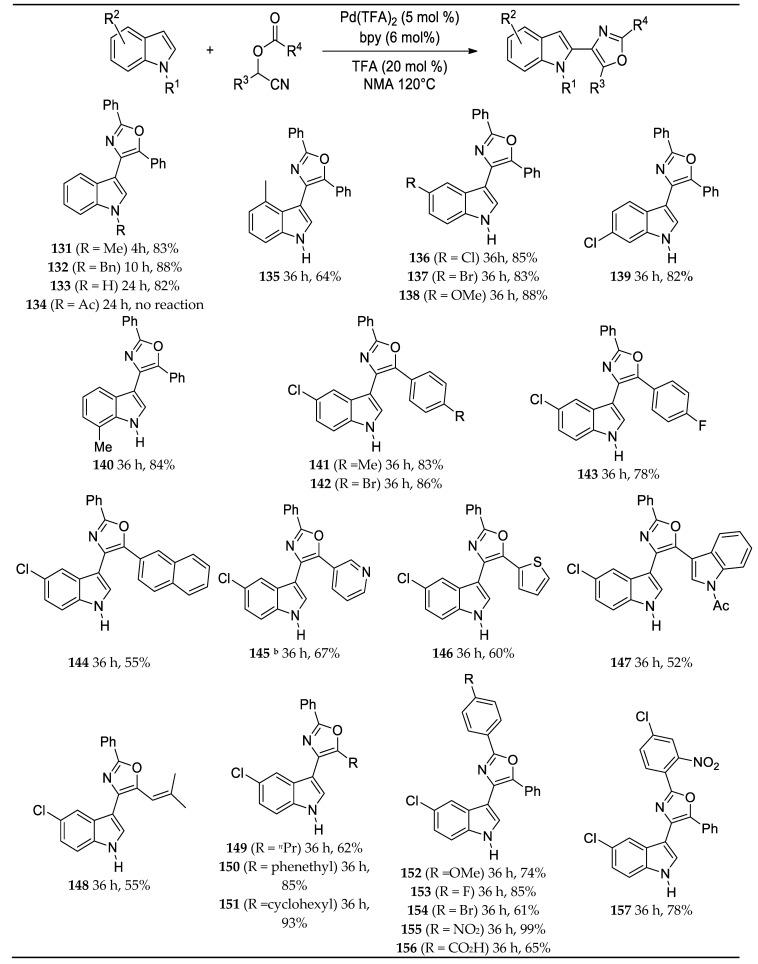

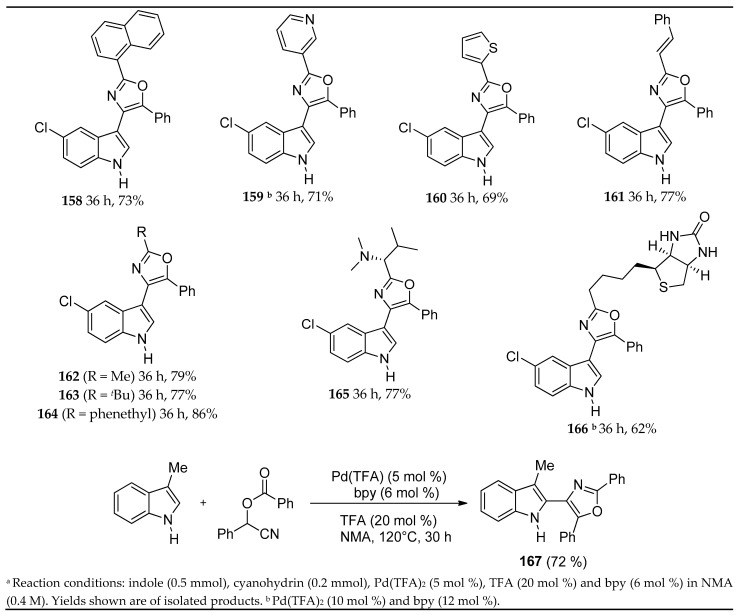
Substrate scope of the reaction between indole derivatives and *O*-acyl cyanohydrins ^a^ [32].

Trisubstituted oxazoles derivatives are synthetized via a Pd-catalyzed direct C-H addition of electron rich aromatic heterocycles to *O*-acylcyanohydrins derived from aldehydes [32]. Optimal reactions conditions include Pd(TFA)_2_ with bipyridine (bpy) as ligand, trifluoroacetic acid (TFA) and N-methylacetamide (NMA) as solvent. Scheme 17 summarizes the scope of the method with indole derivatives as the heterocycle. Good yields of oxazole derivatives are obtained independently of the electronic nature of the substituents (entries 136–138). Both N-H and N-R indoles give the desired products in around 80% yield (entries 131–133). However, when R = Ac no reaction takes place (entry 134).

**Scheme 18:**
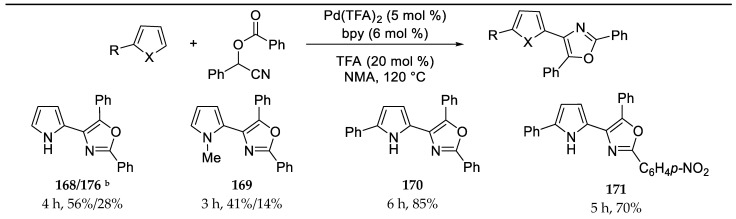

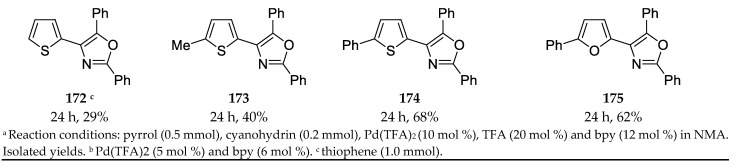
Substrate scope of the reaction between heterocyclic nucleophiles and *O*-acyl cyanohydrins ^a^ [32].

With pyrrole, thiophene and furane derivatives in place of indoles, the reaction affords lower yields of the corresponding oxazoles (Scheme 18) [32].

**Scheme 19:**
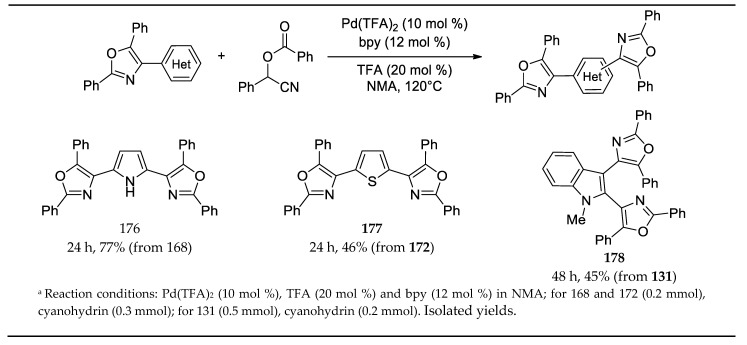
Reaction between oxazole substituted heterocycles and *O*-acyl cyanohydrins ^a^ [32].

This method can also be applied to oxazole substituted heterocycles to produce *bis*-oxazole derivatives in moderate yields. Scheme 19 shows the results when oxazole substituted pyrrole, thiophene and furane are used.

### 3.3. Synthesis of 2-Aminocyclopentanones and 2-Amino-4-Azacyclopentanones

**Scheme 20:**
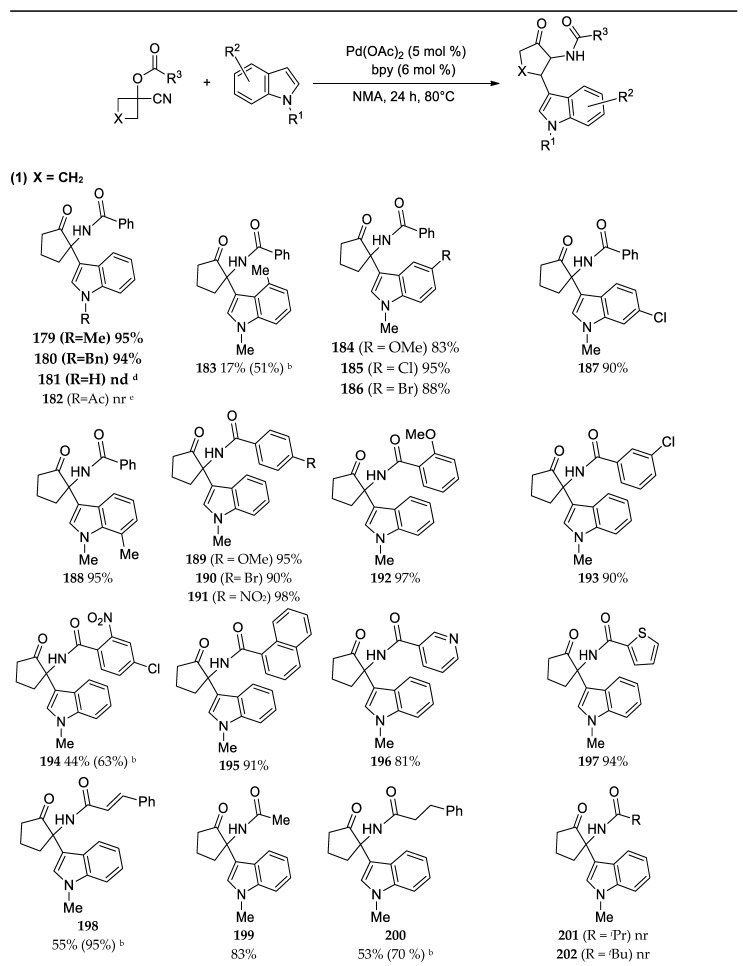

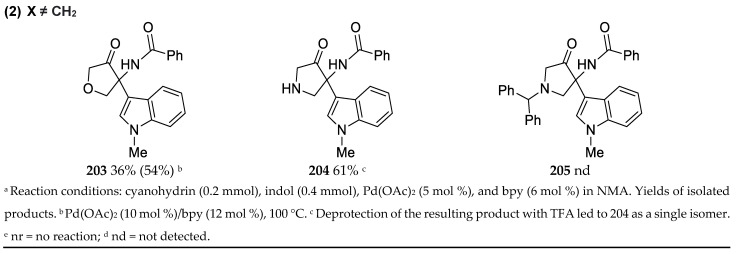
Substrate scope for the reaction between indoles and cyanohydrins ^a^ [33].

Derivatives of 2-aminocyclopentanones and 2-amino-4-azacyclopentanones are obtained through the Pd-catalyzed C-H addition of aromatic heterocycles to the cyano group of *O*-acyl cyanohydrins of cyclobutanone and 3-azacyclobutanone (Scheme 20). The use of Pd(OAc)_2_ with bpy as ligand in NMA at 80 °C are the optimal reaction conditions for this method. *N*-alkyl substituted indoles (**179** and **180**) give high yields of products, while no reaction is observed for *N*-unsubstituted indole (**181**) and when R = Ac (entry **182**). *O*-Benzoyl and *O*-substituted benzoyl protecting cyclobutanone cyanohydrins afford the product in high yields (**183**–**199**). The electronic nature of the substituent has no significant influence on the yield of the reaction (**184**–**186**). With bulky alkyl groups (**201** and **202**) no reaction takes place [33].

### 3.4. Synthesis of Cinnamic Esters

**Scheme 21:**
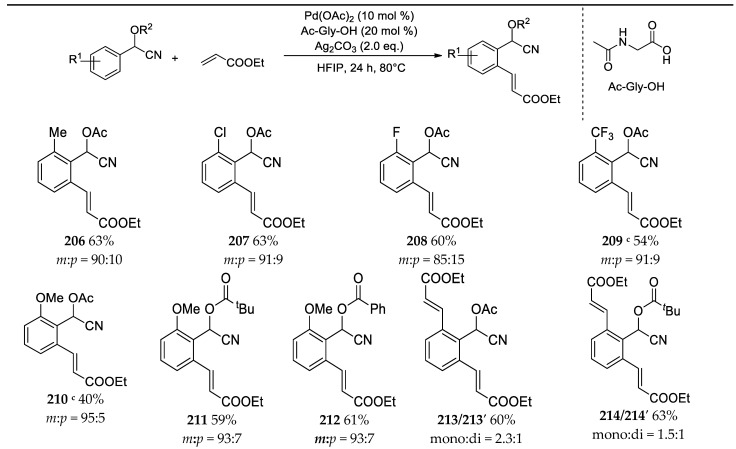

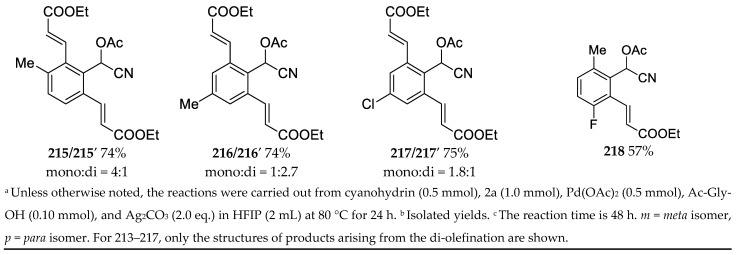
Reaction scope of the Pd-catalyzed C-H olefination of acetyl cyanohydrins ^a,b^ [34].

*Ortho* functionalization of *O*-acetyl cyanohydrins from substituted benzaldehydes has been achieved by a Pd-catalyzed C-H olefination. Optimal reaction conditions involve Pd(OAc)_2_, *N*-acetyl glycine (Ac-Gly-OH) as ligand and AgCO_3_ as oxidant in hexafluoroisopropanol (HFIP). Scheme 21 shows the scope of the reaction. The *O*-acetyl cyanohydrins derived from *ortho*-substituted benzaldehydes affords monoolefination with ethyl acrylate and the reaction functions well independently of the electronic nature of the substituent (**206**–**212**). Mixtures of regioisomers are obtained with preponderance of the *ortho*-olefination. When *ortho*-unsubstituted benzaldehyde is the source of the cyanohydrin mono- and di-olefination substitutions take place in variable ratio (**213**–**217**) [34].

### 3.5. Synthesis of 4-Amino-2(5H)-Furanones

4-Amino-2(5*H*)-furanones are obtained by intramolecular addition of zincates to nitrile group by treatment of *O*-(α-bromoacyl)cyanohydrins. Table 16 shows the scope of the reaction. Highly enantiomerically enriched *O*-(α-bromoacyl)cyanohydrins were used and the reaction proceeds with no or little racemization. Both electron rich and electron deficient substituted (entries 2 and 3) cyanohydrins give good yields of the product [35]. 

### 3.6. Synthesis of Substituted 2-Vinyl-2-Cyclopentenones

**Scheme 22:**
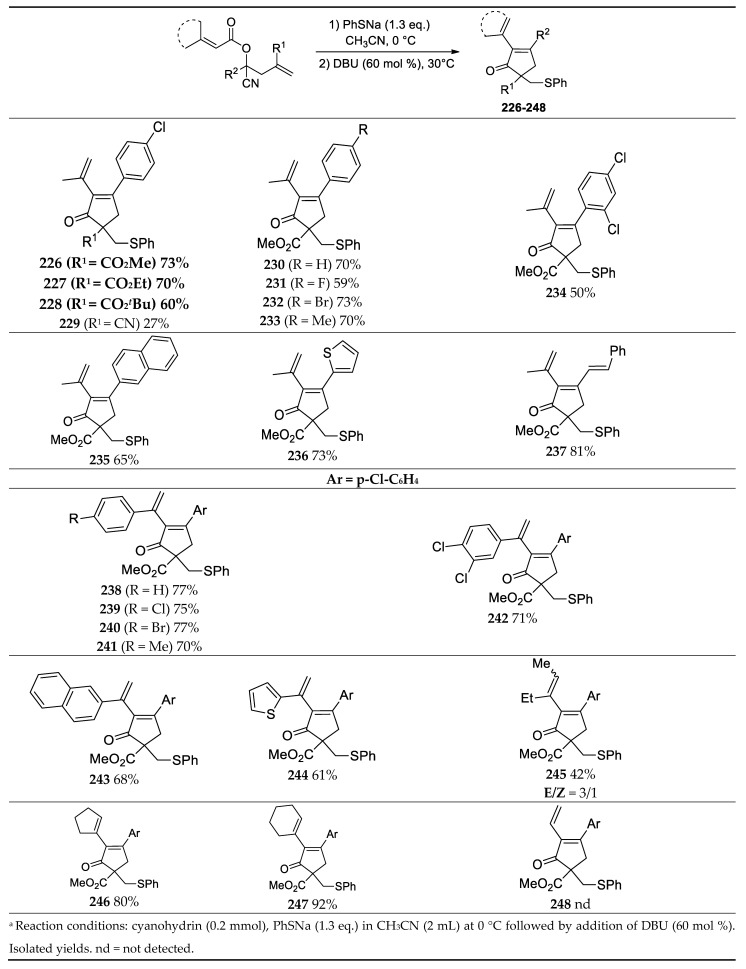
Reaction scope for the synthesis of 2-vinylcyclopentenones by sulfa-Michael addition reaction ^a^ [36].

Highly substituted 2-vinyl-2-cyclopentenones are prepared by a one-pot tandem reaction initiated by a sulfa-Michael addition reaction (SMA) followed by a sequence of two intramolecular aldol reactions and terminating with a dehydroxilation step. Sodium thiophenolate is used as the sulfur nucleophile and DBU as the base. Scheme 22 summarizes the scope of the reaction [36].

### 3.7. Synthesis of O-Acylcyanohydrins from O-(α-Bromoacyl)Cyanohydrins

*O*-acylcyanohydrins with acyl groups larger than acetyl (entries 1–9 in Table 17) can be obtained from *O*-(α-bromoacyl)cyanohydrins by a Pd-catalyzed C-C cross-coupling reaction with boronic acids (Suzuki reaction). Optimal reaction conditions involve Pd(OAc)_2,_ P(*o*-tol)_3_ as ligand in toluene at 60 °C. Table 17 summarizes the results when enantiomerically pure cyanohydrins from benzaldehyde or 3-chlorobenzaldehyde are used. Both electron-withdrawing and electron-donating substituted phenylboronic acid give high yields of the desired products with almost no racemization [37].

**Scheme 23:**
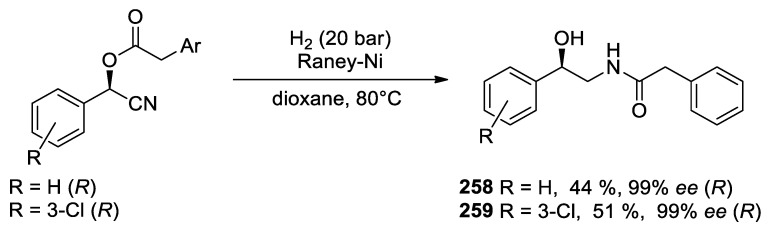
Catalytic hydrogenation of nitriles of acyl cyanohydrins [37].

The *O*-acylated cyanohydrins can give *N*-acylated β-amino alcohols in moderate yields by a catalytic hydrogenation with Raney-Ni (**258** and **259** in Scheme 23).

### 3.8. Synthesis of Substituted Cyclopropylamines and 1,4-Diketones

**Scheme 24:**
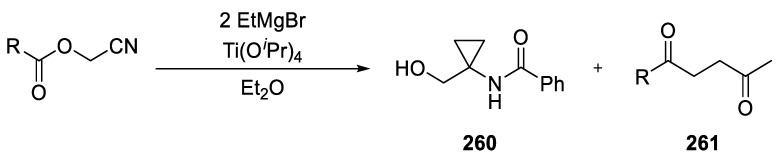
Titanium mediated addition of EtMgBr to *O*-protected cyanohydrins of formaldehyde [38].

*O*-Ethoxycarbonyl cyanohydrin (R = OEt) and *O*-acetyl cyanohydrin (R = CH_3_) of formaldehyde react with EtMgBr/Ti(O*^i^*Pr)_4_ to give substituted cyclopropylamines **260** and 1,4-diketones **261** (Scheme 24) [38,39].

**Scheme 25:**
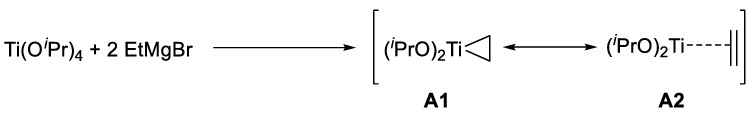
Formation of π-alkene titanium complex from ethylmagnesium bromide.

Ethylmagnesium bromide reacts with titanium(IV)isopropoxide to form diisopropoxyltitanacyclopropane **A1** which isomer is the reactive π-alkene complex **A2** (Scheme 25). 

Table 18 shows the scope of the reaction with various *O*-aroyl and *O*-acyl cyanohydrins. In general, Et_2_O favors the formation of the diketone **261** (entries 1–10, 12–32), whereas THF increases the formation of the cyclopropane **260** maintaining the diketone **261** as the main product. When R = OEt (entry 33), no formation of **261** was observed. The yields of the products are moderate to good. 

### 3.9. Synthesis of α,α-Disubstituted α-Amino-Acids

**Scheme 26:**
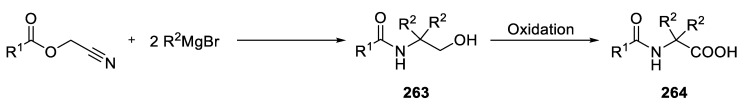
Preparation of *N*-acyl disubstituted amino acids through addition of Grignard reagents followed by oxidation [40].

Symmetrical α,α-disubstituted α-amino-acids **264** are prepared by oxidation of *N*-acyl amino alcohols **263** obtained by a double addition of Grignard reagents to acylcyanohydrins of formaldehyde (Scheme 26) [40].

Table 19 summarizes the scope of the reaction of the addition of Grignard reagent to acylcyanohydrins. In this reaction, two products can be formed depending on the relative reactivity of the cyano or ester moiety towards the Grignard reagent. The amino alcohols **263** is favored when the Grignard reagent adds preferentially to the nitrile group, while the tertiary alcohol **265** is produced when the Grignard derivatives adds to the ester group. Electron-donating groups can deactivate the ester moiety towards addition (entries 3, 5, 6) and steric hindrance of the ester group (entry 7) favors the tertiary alcohol. The solvent plays a crucial role. For instance, in THF, the amino alcohol is produced in preference over the tertiary alcohol (entry 1) but in diethylether, the tertiary alcohol is obtained preferentially (entry 2).

Table 20 summarizes the results of the addition of Grignard reagents to *O*-1-naphtyloylcyanohydrin of formaldehyde. Alkyl (entries 1–4), aryl (entries 5–7) and substituted allyl Grignard compounds react in good yields.

### 3.10. Synthesis of 2-Hydroxy-2-Cyclopentenones

2-Hydroxy-2-cyclopentenones **268** (Table 21) are obtained by reaction of cyanohydrin derivatives with titanacyclopropane in Et_2_O which favors the formation of diketone **267**, which in a further step reacts with a base to produce the cyclopentenone **268** via an intramolecular cyclization. These two transformations can be carried out in one pot by adding to the reaction mixture of the first transformation a degassed NaOH aqueous solution without isolation of the diketone. Table 21 summarizes the results. With the one step method the cyclopentenones **268** are obtained in higher yields than with the two steps method (entries 1–4). Substituted aromatic cyanohydrins (entries 2–4) give better yields the aliphatic cyanohydrins (entries 5–8). Low yield of cyclopentenone is obtained from the cyanohydrin from phenylpropargyl aldehyde (21%, entry 9) [41].

### 3.11. Synthesis of Highly Functionalized Acyclic Ketones

**Scheme 27:**
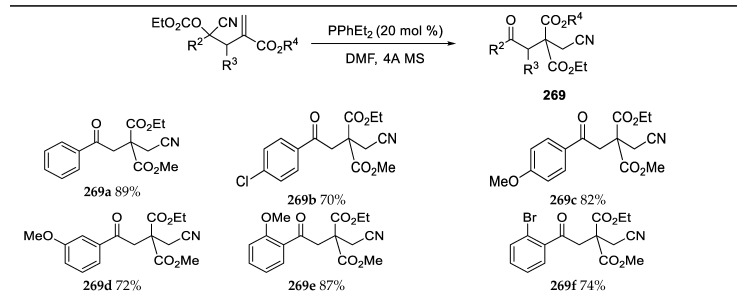

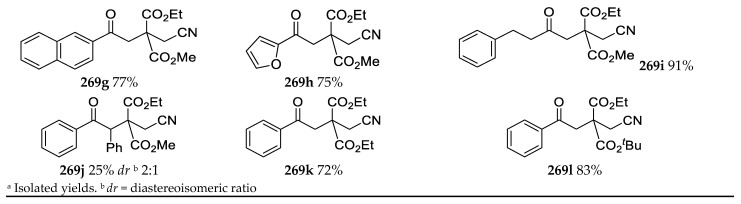
Phosphine promoted intramolecular acylcyanation of various cyanohydrins ^a^ [42].

Highly functionalized acyclic ketones (**269**) have been prepared by Lewis base catalyzed acylcyanation of activated alkenes. Optimal reactions conditions employ 20 mol % of PPhEt_2_ as the Lewis base catalyst in DMF in the presence of molecular sieves. The results are summarized in Scheme 27. Good yields of ketones are obtained regardless of the electronic nature of the substituted phenyl ring [42].

### 3.12. Synthesis of Substituted 1,3-Diketones

Substituted 1,3-diketones **270** are synthetized by DBU as a Lewis base in a rearrangement of allylic *O*-acylcyanohydrins from allylic aromatic ketones. Table 22 shows the scope of this method with *O*-aromatic acylated cyanohydrins. Moderate to good yields are obtained. In all cases where diastereomeric isomers are possible, the diastereoisomeric ratio (*dr*) is approximately 1:1 [43].

Good yields of 1,3-diketones are obtained by this method when *O*-aliphatic acylated cyanohydrins are used. Table 23 shows the scope of this method. With ethyl carbonate of cyanohydrin (R^1^ = EtO) no reaction is observed (entry 3). No diastereoselectivity is observed in this reaction (*dr* = 1:1 in most cases).

### 3.13. Synthesis of 2,4,5-Trisubstituted Oxazoles by Palladium Catalyzed C-H Activation

**Scheme 28:**
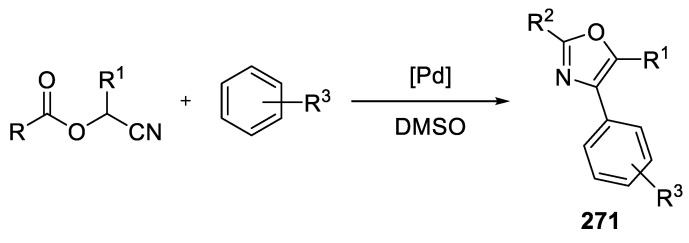
Synthesis of trisubstituted oxazoles by palladium-catalyzed C-H activation of arenes [44].

**Scheme 29:**
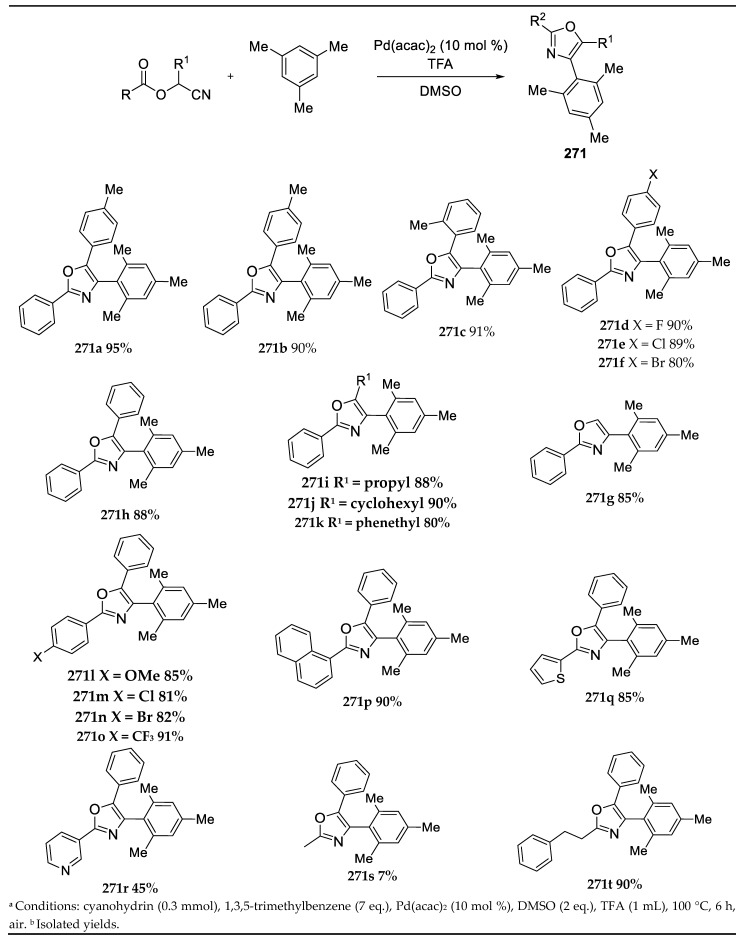
Scope of the use of functionalized nitriles in the preparation of substituted oxazoles ^a,b^ [44].

2,4,5-trisubstituted oxazoles can be obtained in one pot by a palladium catalyzed C-H activation of arenes followed by carbopalladation and an annulation sequence (Scheme 28). Optimal results are found with Pd(acac)_2_ with TFA in DMSO and no oxidant is necessary. As shown in Scheme 29, *O*-aroylcyanohydrins give high yields of trisubstituted oxazoles with electron rich 1,3,5-trimethylbenzene. Cyanohydrins from aromatic or aliphatic aldehydes behave similarly. The reaction is not sensitive to the electronic nature of the *O*-aroyl group (**271l**–**271o**). When R^2^ = 3-pyridyl, the yield lowers to 45% [44].

**Scheme 30:**
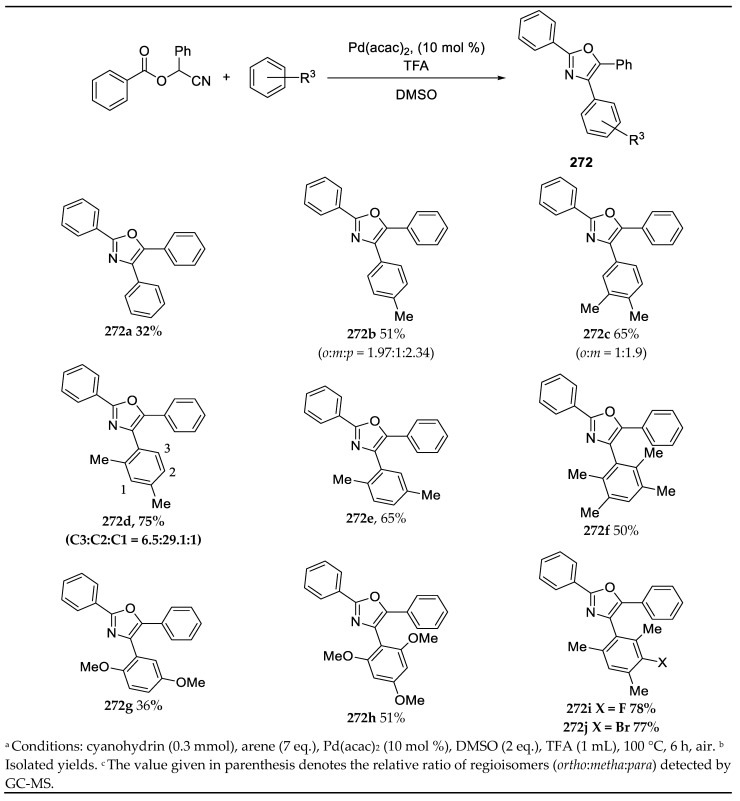
Use of simple arenes in the preparation of substituted oxazoles ^a,b,c^ [44].

Scheme 30 shows the results with other arenes. With benzaldehyde, the yield dropped to 32%. A more nucleophilic arene like toluene raises the yield to 51% of a mixture of regioisomers and with 1,2-dimethylbenzene, the yield increases to 65%. These observations imply that the reaction requires electron-rich arenes. This method is also sensitive to steric hindrance of the arene even with electron-donating groups (**272g**, **272h**).

**Scheme 31:**
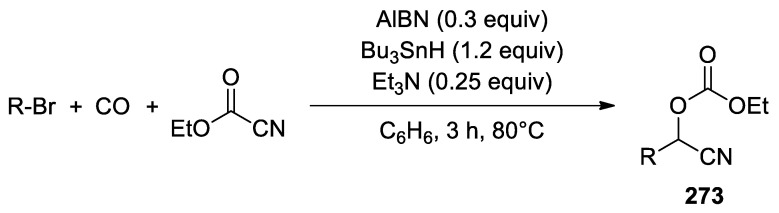
Three components coupling reaction to cyanohydrin derivatives [45].

So far, all the methods for the preparation of *O*-ethoxycarbonyl/acetyl cyanohydrins discussed employed aldehydes or ketones as the starting material. Additionally, alkyl halides can be used in the synthesis of cyanohydrins by means of a radical formylation reaction. A one-pot synthesis of *O*-ethoxycarbonyl cyanohydrins from alkyl bromides via radical formylation of the alkyl bromide with CO at high pressure followed by a nucleophilic addition of cyanide ion has been developed (Scheme 31). AIBN is used to induce radical formylation of the alkyl bromide. This method involves two one-carbon components which increase the carbon chain of the alkyl bromide in two carbon units [45].

Table 24 shows the scope of the method. The reaction tolerates various functional groups like Cl (entry 2), ethoxycarbonyl (entry 3) and CN (entry 4), primary bromides (entries 1–5 and 10), secondary bromides (entries 6–8) and tertiary bromides (entry 9).

## 4. Conclusions

The synthetic importance of *O*-ethoxycarbonyl and acylcyanohydrins continues to make the discovery of new methods for their preparation a thriving field of research. In particular, alkalicyanides and alkylcyanoformates are considered major cyanide ion sources. Surfactants, ionic liquids, organocatalysts, transition-metal catalysts with chiral ligands are some of the strategies used to develop more specific, efficient, and greener processes. Taking advantage of the specific reactivity of the protected group, in addition to the intrinsic reactivity of the cyanohydrin, can allow the preparation of an almost endless variety of interesting synthons such as highly substituted cyclohexenes, oxazoles, cyclopentenones, cynamic esteres, furanones, among others. As such, new powerful synthetic methods and applications of *O*-ethoxycarbonyl and *O*-acyl cyanohydrins are likely to arise in the near future.

## Figures and Tables

**Table 1:** Cyanocarbonation of aldehydes ^a^ [19].

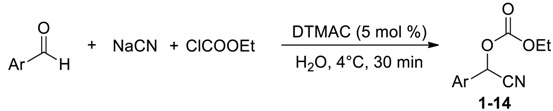			
Entry	Aldehyde	Product	Yield (%) ^b^
**1**	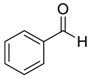	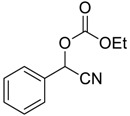 **1**	98
**2**	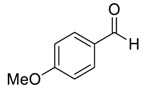	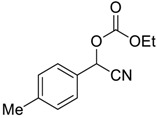 **2**	96
**3**	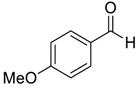	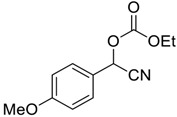 **3**	94
**4**	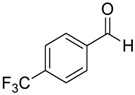	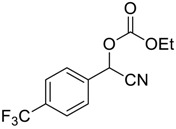 **4**	97
**5**	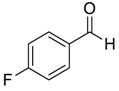	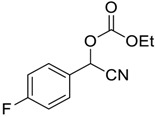 **5**	97
**6**	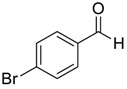	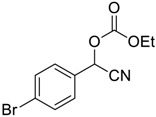 **6**	96
**7**	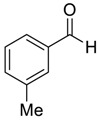	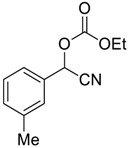 **7**	97
**8**	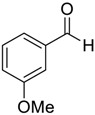	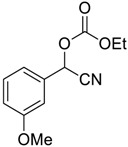 **8**	96
**9**	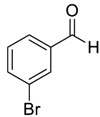	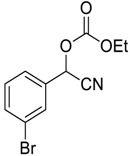 **9**	98
**10**	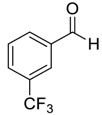	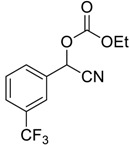 **10**	97
**11**	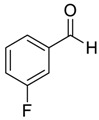	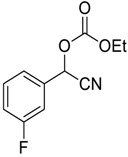 **11**	97
**12**	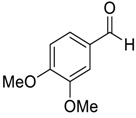	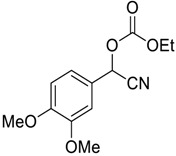 **12**	95 ^c^
**13**	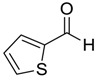	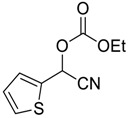 **13**	96
**14**	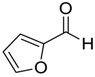	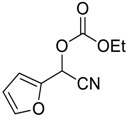 **14**	97

^a^ Reaction conditions: aldehyde (30 mmol), ethyl chloroformate (36 mmol), and DTAC (5 mol %) in H_2_O (1 mL) were stirred at 4 °C and NaCN (36 mmol) in H_2_O (1 mL) were added to the reaction mixture. ^b^ Isolated yields. ^c^ The aldehyde was dissolved in 3 mL of THF.

**Table 2:** Cyanoethoxycarbonilation of aldehydes in ionic liquids ^a^ [19].

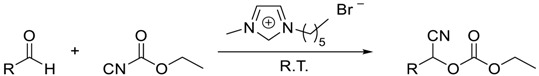				
Entry	Substrate	Product	Conversion (%) ^b^	Yield (%) ^c^
**1**	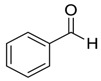	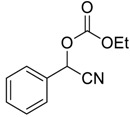 **1**	>98	94
**2**	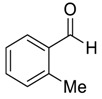	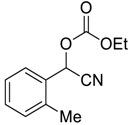 **15**	>98	95
**3**	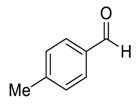	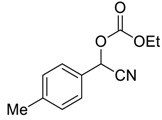 **2**	>98	93
**4**	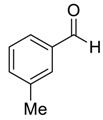	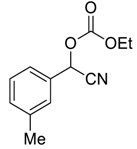 **7**	>99	95
**5**	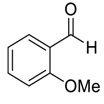	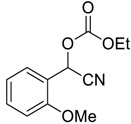 **16**	>99	96
**6**	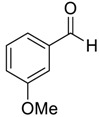	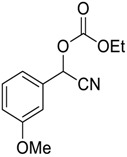 **8**	>98	92
**7**	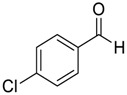	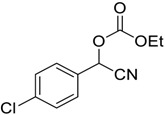 **17**	>98	93
**8**	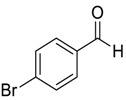	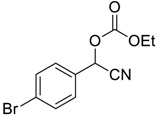 **6**	>99	95
**9**	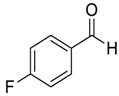	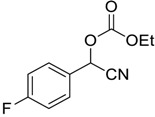 **5**	>99	93
**10**	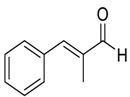	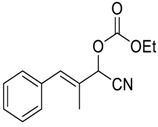 **18**	>98	94
**11**	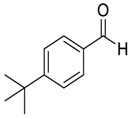	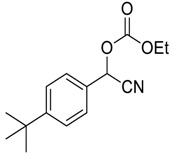 **19**	>97	91
**12**	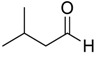	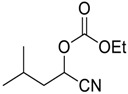 **20**	>97	90
**13**	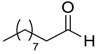	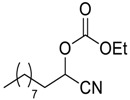 **21**	>98	92

^a^ 0.5 g (2.3 mmol) of ionic liquid, 0.625, mmol of aldehydes and 1.2 mmol of ethylcyanoformate were stirred for 2 h at room temperature. ^b^ Conversion determined by GC. ^c^ Isolated yields.

**Table 3:** Cyanoethoxycarbonilation of aldehydes catalyzed by DMAP under solvent free conditions ^a^ [22].

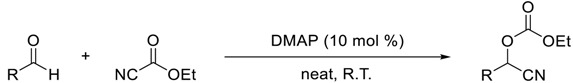				
Entry	Aldehyde	Product	Time (min)	Yield (%) ^b^
**1**	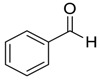	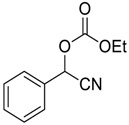 **1**	90	89
**2**	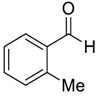	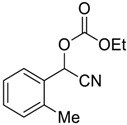 **15**	90	90
**3**	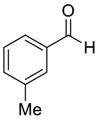	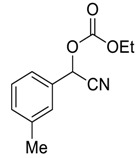 **7**	90	91
**4**	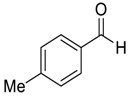	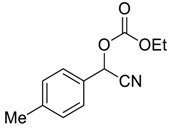 **3**	90	87
**5**	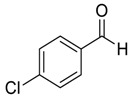	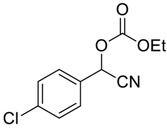 **17**	480	62
**6**	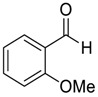	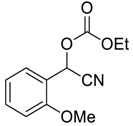 **16**	40	94
**7**	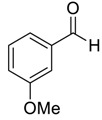	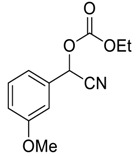 **8**	40	93
**8**	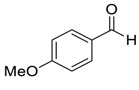	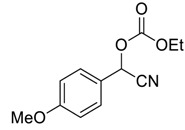 **3**	40	92
**9**	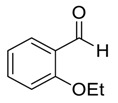	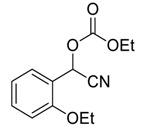 **22**	40	94
**10**	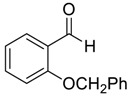	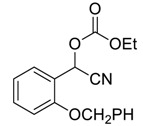 **23**	40	95
**11**	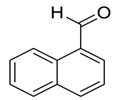	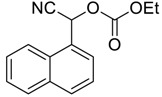 **24**	15	93
**12**	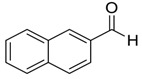	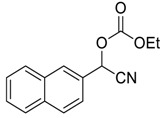 **25**	15	93
**13**	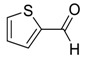	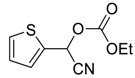 **13**	35	91
**14**	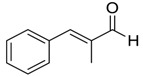	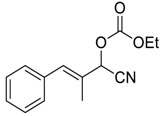 **18**	60	92
**15**	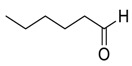	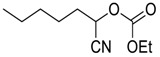 **26**	60	72
**16**	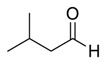	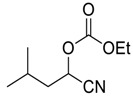 **20**	60	86
**17**	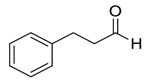	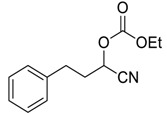 **27**	180	80

^a^ All reactions were carried out using aldehyde (1 mmol), ethylcyanoformate (1.5 mmol), in the presence of 10 mol % DMAP at room temperature. ^b^ Isolated yields.

**Table 4:** Cyanation of aldehydes with ethyl cyanoformate catalyzed by DMAP ^a^ [23].

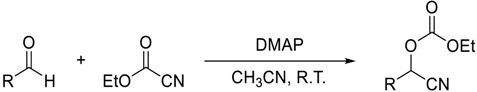					
Entry	DMAP mol %	Aldehyde	Product	Time (h)	Yield (%) ^b^
**1**	1	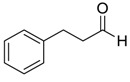	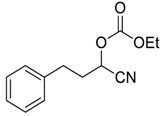 **27**	0.5	96
**2**	1	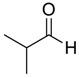	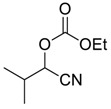 **28**	1	83
**3**	1	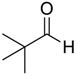	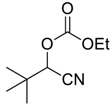 **29**	2	55
**4**	5	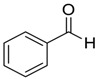	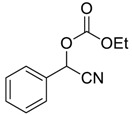 **1**	8	99
**5**	5	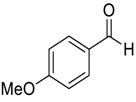	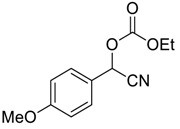 **3**	24	81
**6**	5	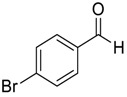	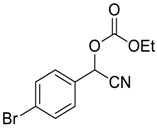 **6**	2	98
**7**	5	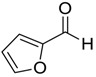	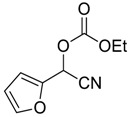 **14**	8	97
**8**	5	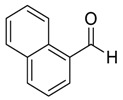	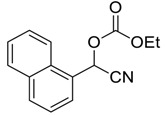 **25**	6	97
**9**	5	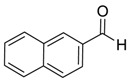	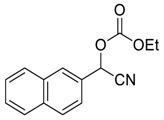 **30**	8	99
**10 ^c,d^**	5	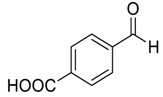	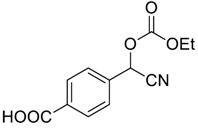 **31**	24	78

^a^ Unless otherwise noted, reactions were carried out by addition of ethyl cyanoformate (0.55 mol) to a solution of aldehyde (0.5 mmol) and DMAP in CH_3_CN (1 mL) at room temperature. ^b^ Isolated yields. ^c^ The reaction was conducted using 2.5 equivalents of ethyl cyanoformate. ^d^ EtOH (3 mL) was used instead of acetonitrile.

**Table 5:** Cyanation of ketones with ethyl cyanoformate catalyzed by DMAP ^a^ [23].

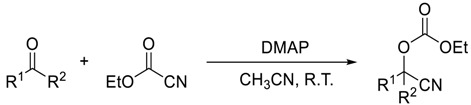				
Entry	Ketone	Product	Time (h)	Yield ^b^ (%)
**1 ^c^**	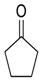	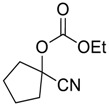 **32**	24	20
**2**	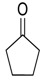	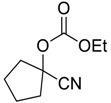 **32**	24	85
**3**	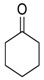	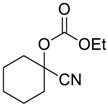 **33**	24	99
**4**	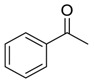	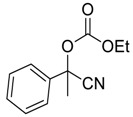 **34**	24	30
**5**	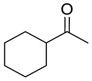	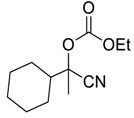 **35**	24	87
**6**	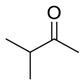	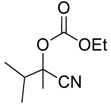 **36**	24	73
**7**	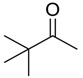	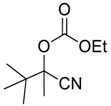 **37**	48	44

^a^ Unless otherwise noted, reactions were carried out by addition of ethyl cyanoformate (0.55 mol) to a solution of ketone (0.5 mmol) and DMAP (10 mol %) without solvent at room temperature. ^b^ Isolated yields. ^c^ The reaction was conducted in acetonitrile (1 mL).

**Table 6:** One-pot synthesis of *O*-acetyl cyanohydrins from aldehydes via *O*-silylcyanohydrins in [bmim]BF_4_^a^ [24].

Entry	Aldehyde	Product	Yield (%) ^b^
**1**	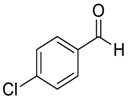	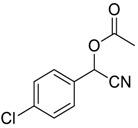 **38**	96
**2**	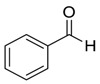	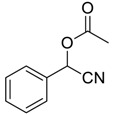 **39**	99
**3**	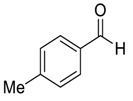	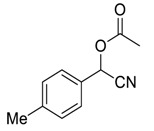 **40**	91
**4**	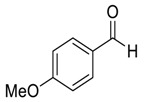	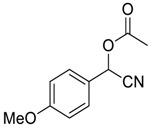 **41**	92
**5**	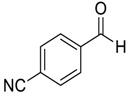	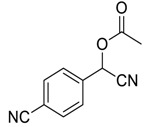 **42**	79
**6**	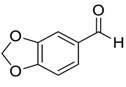	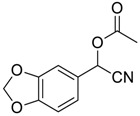 **43**	91
**7**	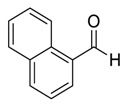	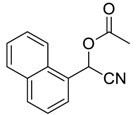 **44**	83
**8**	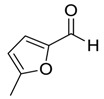	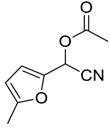 **45**	93
**9**	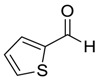	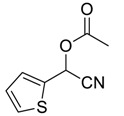 **46**	76
**10**	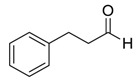	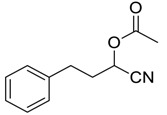 **47**	87

^a^ Reaction conditions: [bmim]BF_4_ (80.5 mL), aldehyde (1.0 mmol), and TMSCN (0.5 mmol) were stirred at room temperature for 12 h. Ac_2_O was added and stirring continues for 12 h. ^b^ Isolated yields.

**Table 7:** Synthesis of *O*-acyl cyanohydrins with TMSCN, acetic anhydride and aldehydes catalyzed by B(C_6_F_5_)_3_^a^ [25].

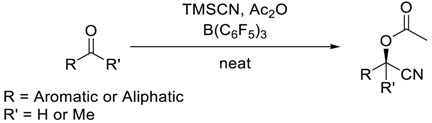				
Entry	Aldehyde	Product	Time (h)	Yield (%) ^b^
**1**	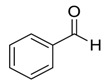	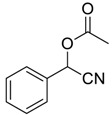 **39**	3	92
**2**	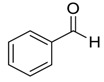	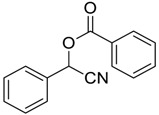 **48**	5	88 ^c^
**3**	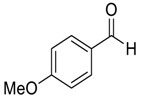	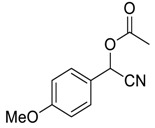 **41**	3	85
**4**	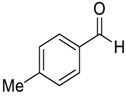	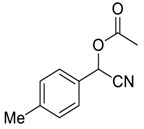 **40**	3	94
**5**	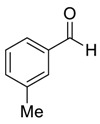	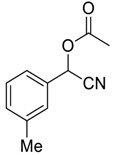 **49**	3	90
**6**	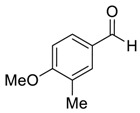	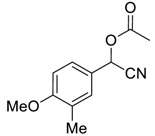 **50**	3	98
**7**	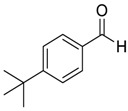	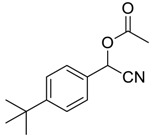 **51**	3	89
**8**	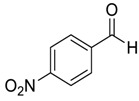	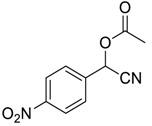 **52**	8	71
**9**	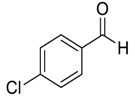	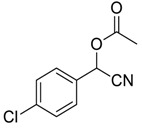 **38**	3	95
**10**	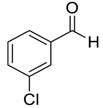	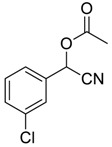 **53**	3	91

^a^ Reagents and conditions: aldehyde (1 mmol), TMSCN (1.2 mmol), B(C_6_F_5_)_3_ (1 mol %) and Ac_2_O (2 mmol) were employed at r.t. ^b^ Isolated yields. ^c^ Benzoyl chloride was used instead of Ac_2_O.

**Table 8:** Synthesis of cyanohydrin esters from aroyl chlorides ^a^ [26].

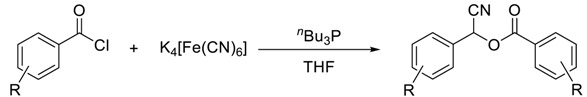			
Entry	Aroyl Chloride	Product	Yield (%) ^b^
**1**	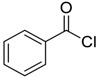	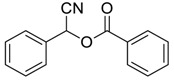 **48**	88
**2**	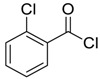	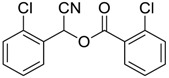 **54**	80
**3**	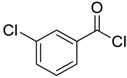	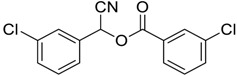 **55**	82
**4**	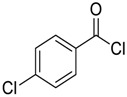	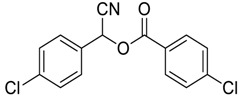 **56**	86
**5**	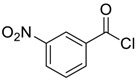	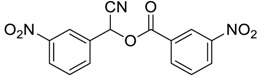 **57**	82
**6**	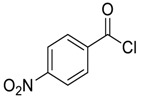	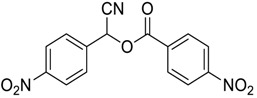 **58**	87
**7**	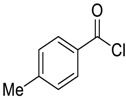	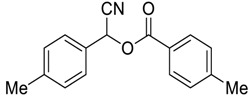 **59**	79
**8**	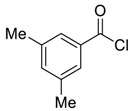	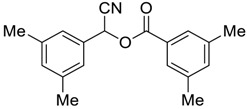 **60**	75
**9**	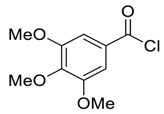	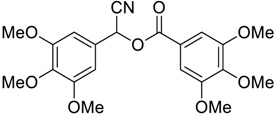 **46**	73
**10**	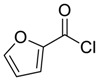	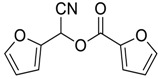 **61**	78

^a^ All reactions were carried out first using benzoyl chloride (10 mmol) and K_4_[Fe(CN)_6_] (2.5 mmol) at 160 °C, then at refluxing conditions after addition of *^n^*Bu_3_P (10 mmol) and THF (10 mL). ^b^ Isolated yields.

**Table 9:** Asymmetric cyanosilylation of aldehydes catalyzed by a thiourea derivative and conversion to *O*-acetylcyanohydrins ^a^ [27].

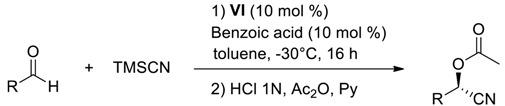				
Entry	Aldehyde	Product	Yield (%) ^b^	*ee* (%) ^c^
**1**	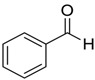	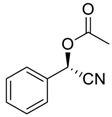 **62**	73	73
**2**	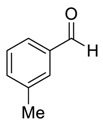	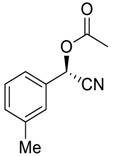 **63**	74	46
**3**	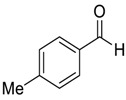	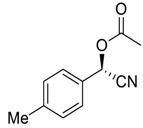 **64**	80	84
**4**	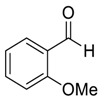	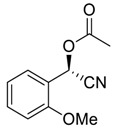 **65**	69	8
**5**	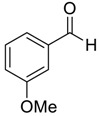	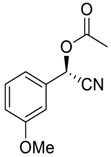 **66**	72	58
**6 ^d^**	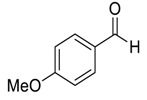	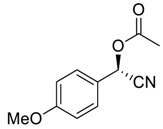 **67**	57	68
**7 ^e^**	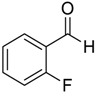	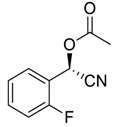 **68**	83	42
**8**	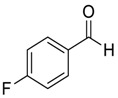	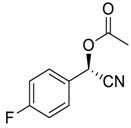 **69**	79	82
**9**	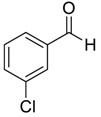	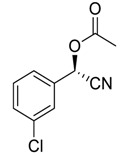 **70**	66	65
**10**	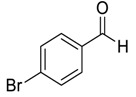	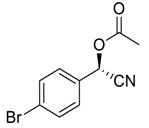 **71**	78	85
**11**	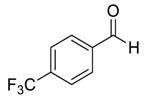	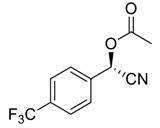 **72**	87	82
**12**	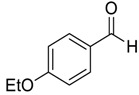	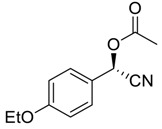 **73**	80	77
**13**	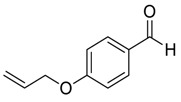	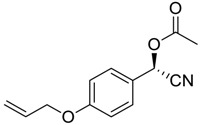 **74**	88	83
**14 ^e^**	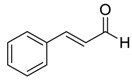	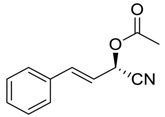 **75**	67	88
**15**	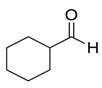	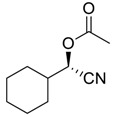 **76**	90	18
**16**	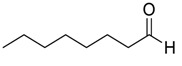	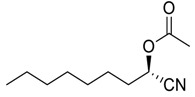 **77**	72	51

^a^ Unless otherwise stated the reactions were carried out on a 0.2 mmol scale in the presence of 10 mol % of **VI** and 10 mol % of benzoic acid with 15 eq. of TMSCN in anhydrous toluene at −30 °C for 16 h. ^b^ Yields of isolated product after the conversion to cyanohydrin acetate. ^c^ Determined by chiral GC or chiral HPLC analysis for the corresponding acetate. ^d^ Reaction time 60 h. ^e^ Reaction time 48 h.

**Table 10:** Asymmetric acetylcyanation of aldehydes catalyzed by vanadium (V) complexes ^a^ [27].

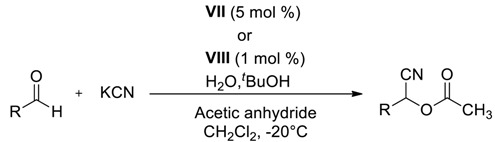						
Entry	Substrate	Product	Catalyst VII		Catalyst VIII	
			Yield (%) ^b^	*ee* (%) ^c^	Yield (%) ^b^	*ee* (%) ^c^
**1**	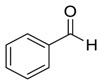	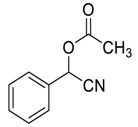 **1**	97	83	99 (98)	92 (90)
**2**	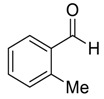	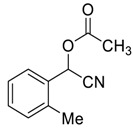 **15**	98	89	98 (99)	>99 (96)
**3**	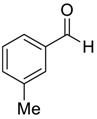	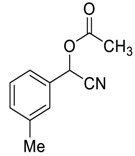 **7**	97^d^	82	95 (95)	91 (88)
**4**	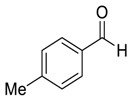	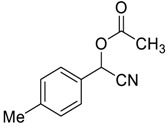 **2**	95	81	95 (93)	90 (89)
**5**	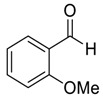	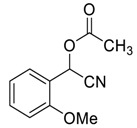 **16**	96	86	95 (96)	97 (95)
**6**	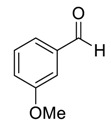	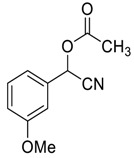 **8**	95	84	99 (99)	96 (95)
**7**	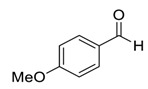	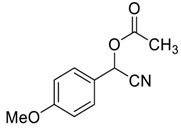 **3**	94	82	97 (94)	96 (94)
**8**	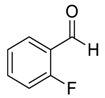	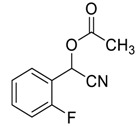 **78**	99	87	97 (96)	>99 (97)
**9**	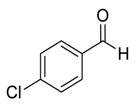	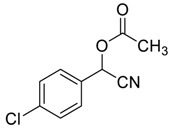 **17**	98	84	97 (94)	92 (90)
**10**	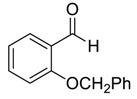	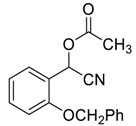 **23**	98	78	97 (96)	89 (85)
**11**	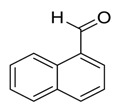	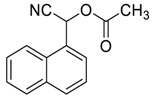 **25**	99	85	99 (99)	>99 (97)
**12**	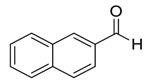	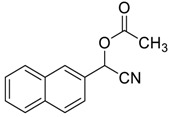 **30**	99	89	99 (99)	>99 (98)
**13**	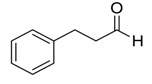	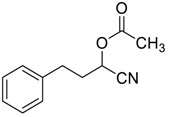 **27**	98	65	96 (95)	78 (76)
**14**	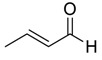	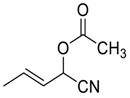 **79**	98	82	98 (98)	89 (85)
**15**	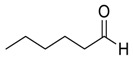	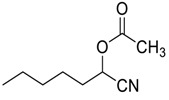 **26**	98	53^d^	99 (97)	73 ^d^ (72)

**^a^** Reaction conditions: catalyst **VII** (5 mol %) or **VIII** (1 mol %), dichloromethane (2 mL), aldehyde (1.2 mmol), KCN (2.4 mmol), H_2_O (1.11 mmol), *^t^*BuOH (82.09 mmol), acetic anhydride (4.8 mmol) at −20 °C in 5–6 h. ^b^ Isolated yields. Data in the parentheses are with NaCN as a cyanide source. ^c^ *ee* were determined by HPLC on chiral OD or AD column. The absolute configuration (*S*) was established by comparison of the optical values with that in the literature. ^d^ *ee* was determined by GC on chiral GTA column.

**Table 11:** Substrate scope of the asymmetric catalytic formation of cyanohydrin carbonates with complex **VII** in the presence of lutidine ^a^ [28].

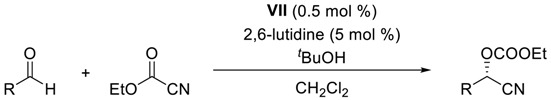					
Entry	Substrate	Time (h)	Product	Yield ^b^ (%)	*ee*^c,d^ (%)
**1**	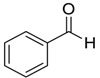 **48**	12	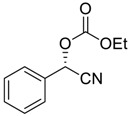 **80**	96	95
**2**	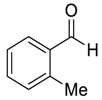 **49**	12	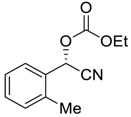 **81**	97	93
**3**	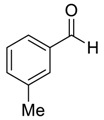 **50**	15	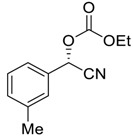 **82**	94	85
**4**	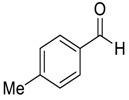 **51**	12	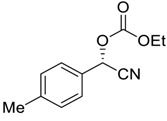 **83**	97	96
**5**	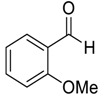 **52**	16	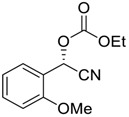 **84**	95	92
**6**	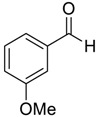 **53**	18	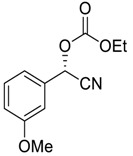 **85**	90	87
**7**	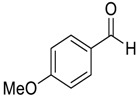 **54**	12	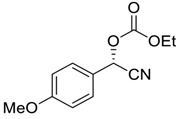 **86**	96	97
**8**	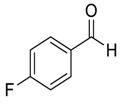 **56**	16	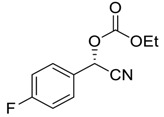 **87**	95	93
**9**	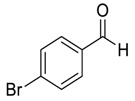 **58**	18	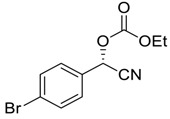 **88**	93	91
**10**	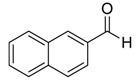 **61**	12	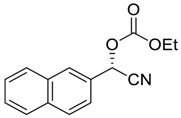 **89**	95	95
**11**	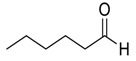 **64**	15	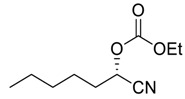 **90**	88	81

^a^ Reaction conditions: **VII** (0.5 mol %), benzaldehyde (1.2 mmol), ethylcyanoformate (1.8 mmol), 2,6,-lutidine (5 mol %), at −20 °C, in 0.8 mL CH_2_Cl_2_. ^b^ Isolated yields. ^c^ *ee* were determined by HPLC on chiral OD, OD-H columns. ^d^ *ee* was determined by chiral GC using chiral GTA column.

**Table 12:** Enantioselective cyanoformylation of aldehydes catalyzed by the Ti(O*^i^*Pr)_4_/**IX** system ^a^ [29].

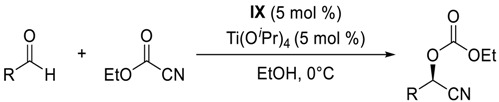					
Entry	Substrate	Product	Time (h)	Yield (%) ^b^	*ee* (%) ^c^
**1**	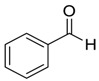	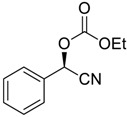 **91**	6	93	83
**2**	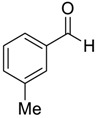	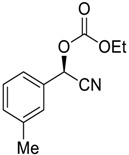 **92**	6	91	77
**3**	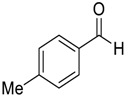	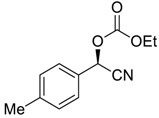 **93**	6	92	75
**4**	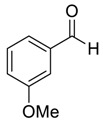	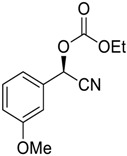 **94**	6	94	71
**5**	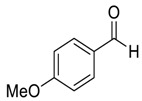	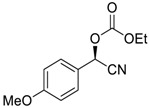 **95**	6	93	73
**6**	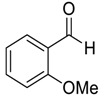	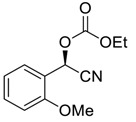 **96**	6	90	85
**7**	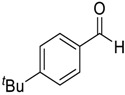	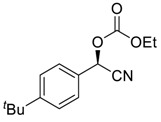 **97**	6	91	75
**8**	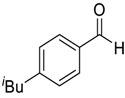	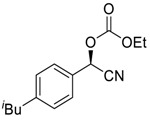 **98**	6	92	71
**9**	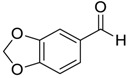	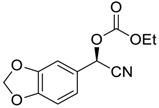 **99**	10	94	65
**10 ^d^**	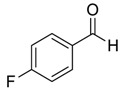	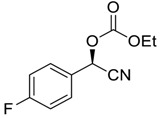 **100**	12	95	71
**11 ^d^**	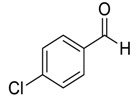	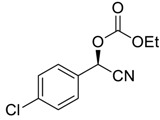 **101**	12	95	63
**12 ^e^**	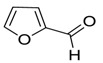	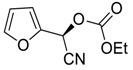 **102**	10	96	73
**13 ^e^**	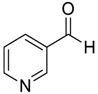	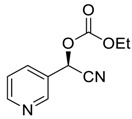 **103**	10	93	61
**14**	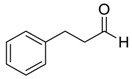	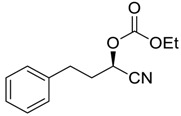 **104**	6	91	67
**15 ^e^**	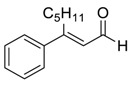	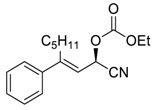 **105**	10	90	71

^a^ All reactions were performed with aldehyde (80.25 mmol), and NCCOOEt (0.375 mmol) in ethanol (2 mL) at 0 °C unless otherwise indicated. ^b^ Isolated yields. ^c^ Determined by HPLC on a Chiralcel OD-H or Chiralcel AD-H column. ^d^ Reaction temperature −50 °C. ^e^ −20 °C.

**Table 13:** Enantioselective cyanation of aldehydes catalyzed by alumminium complex ^a^ [30].

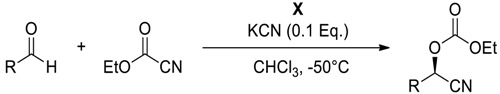						
Entry	Substrate	Product	Catalyst (mol %)	Time (h)	Yield (%) ^b^	*ee* (%) ^c^
**1**	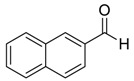	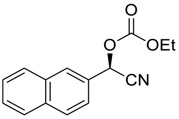 **106**	0.1	24	>99	93
**2**	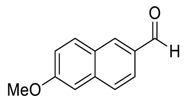	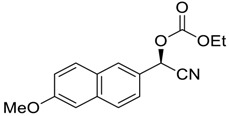 **107**	0.1	24	92	93
**3**	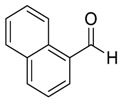	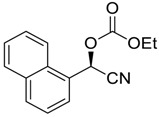 **108**	0.1	24	80	79
**4**	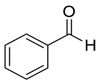	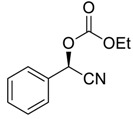 **91**	0.1	24	99	91
**5**	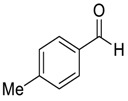	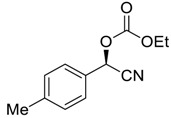 **93**	0.1	72	85	88
**6**	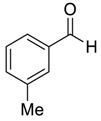	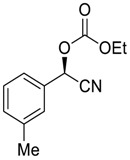 **92**	0.1	48	98	90
**7**	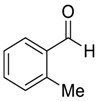	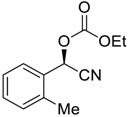 **109**	0.1	48	99	82
**8**	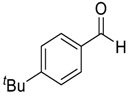	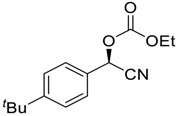 **97**	0.5	72	51	85
**9**	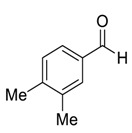	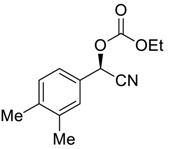 **110**	0.1	72	83	86
**10**	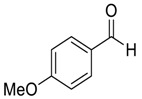	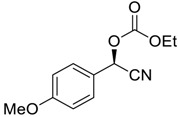 **95**	0.5	72	78	93
**11**	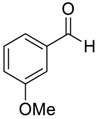	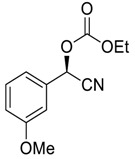 **94**	0.1	24	>99	92
**12**	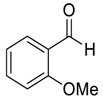	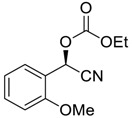 **96**	0.1	48	61	84
**13**	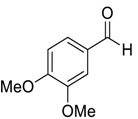	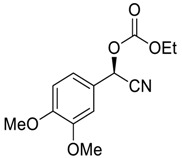 **111**	0.5	72	97	96
**14**	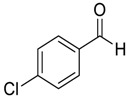	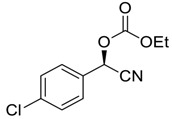 **101**	0.1	48	>99	89
**15**	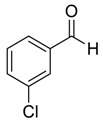	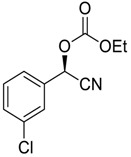 **112**	0.1	24	93	79
**16**	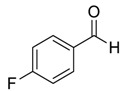	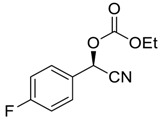 **100**	0.1	48	>99	89
**17**	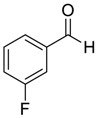	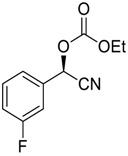 **113**	0.1	24	98	78
**18**	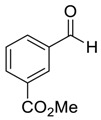	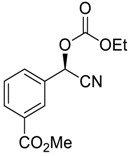 **114**	0.1	48	99	80
**19**	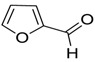	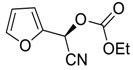 **102**	0.1	48	98	82
**20**	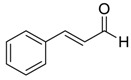	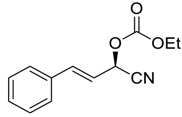 **115**	0.1	48	>99	96
**21**	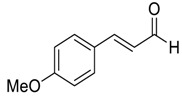	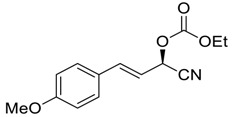 **116**	0.1	48	90	97
**22 ^d^**	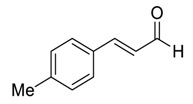	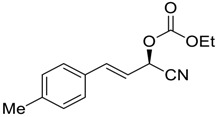 **117**	0.1	72	96	94
**23 ^e^**	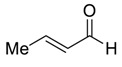	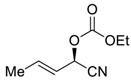 **118**	0.01	72	>99	93
**24 ^d^**	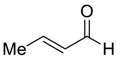	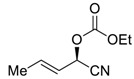 **118**	0.1	48	>99	80
**25 ^d^**	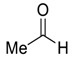	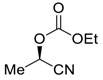 **119**	0.1	72	99	81
**26 ^d^**	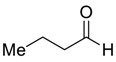	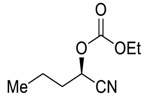 **120**	0.1	72	89	78
**27 ^d^**	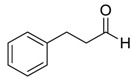	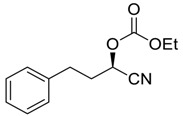 **104**	0.1	72	99	90
**28 ^d^**	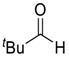	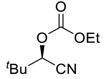 **121**	0.1	72	99	78

^a^ Reactions conditions: To a mixture of catalyst {Al-F}OTf and KCN (0.1 eq.) in CHCl_3_, aldehyde (1.0 eq.) and ethyl cyanoformate (1.0 eq.) were added at −50 °C. The mixture was stirred 24 h at −50 °C. ^b^ Isolated yields after column chromatography. ^c^ Determined by HPLC. ^d^ Reaction in CH_2_Cl_2_/CHCl_3_ (1:1) at −80 °C. ^e^ 0.05 eq. of KCN.

**Table 14:** Investigation of the substrate scope for the carboxycyanation with pyrocarbonate and KCN ^a^ [30].

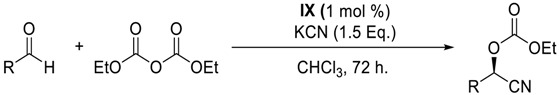					
Entry	Substrate	Product	Temperature (°C)	Yield (%) ^b^	*ee* (%) ^c^
**1**	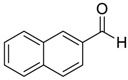	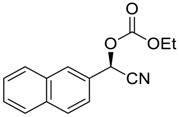 **106**	−60	96	93
**2**	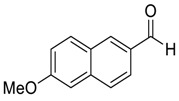	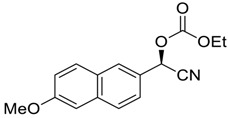 **107**	−60	93	93
**3 ^c^**	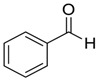	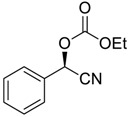 **91**	−60	92	88
**4 ^d^**	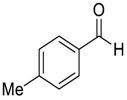	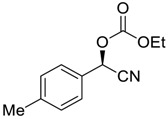 **93**	−80	85	90
**5**	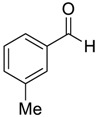	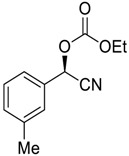 **92**	−60	98	91
**6**	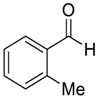	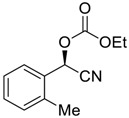 **109**	−60	>99	82
**7 ^f^**	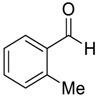	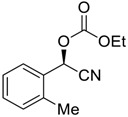 **109**	−60	98	90
**8**	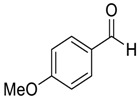	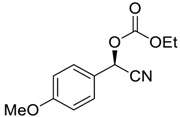 **95**	−60	99	93
**9**	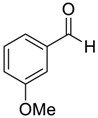	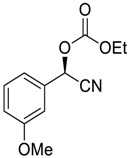 **94**	−60	>99	85
**10**	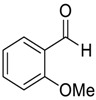	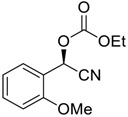 **96**	−60	>99	90
**11**	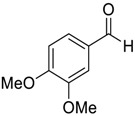	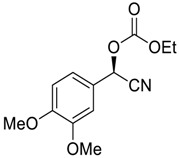 **111**	−60	90	96
**12**	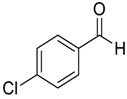	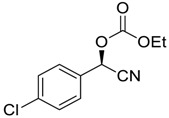 **101**	−60	>99	80
**13**	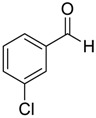	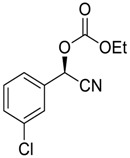 **112**	−60	>99	80
**14**	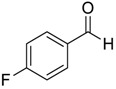	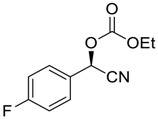 **100**	−60	>99	80
**15**	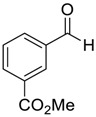	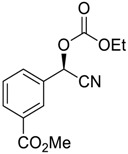 **114**	−60	92	82
**16 ^d^**	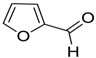	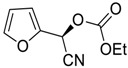 **102**	−80	93	90
**17**	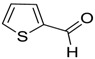	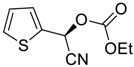 **122**	−60	91	80
**18**	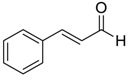	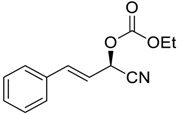 **115**	−60	>99	92
**19**	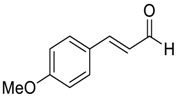	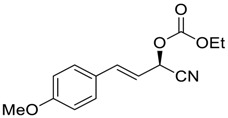 **116**	−60	99	93
**20 ^e^**	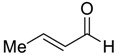	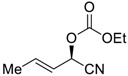 **118**	−60	90	95
**21 ^f^**	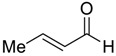	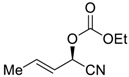 **118**	−60	81	94
**22**	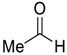	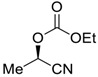 **119**	−60	>99	55
**23 ^d^**	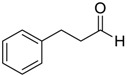	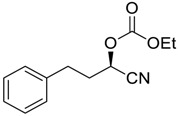 **104**	−80	95	69

^a^ Reactions conditions: To a mixture of catalyst {Al-F}OTf (1 mol %) in CHCl_3_ the aldehyde (1.0 eq.) was added at −60 °C. Then (EtO(CO))_2_O (4 eq.) was added and the mixture was stirred for 72 h at −60 °C. ^b^ Yield of isolated product after column chromatography. ^c^ Determined by HPLC. ^d^ Reaction in CH_2_Cl_2_/CHCl_3_ (1:1). ^e^ 0.5 mol % of catalyst. ^f^ 0.1 mol % of catalyst.

**Table 15:** Synthesis of compounds **123–130** by addition of anions of ethyl carbonates of cyanohydrins to 2-cycloalkenones ^a^ [31].

Entry	Cyanohydrin	Product	Yield (%) ^b^
**1**	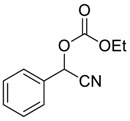 **1**	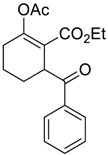 **123**	77
**2**	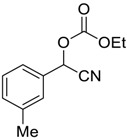 **7**	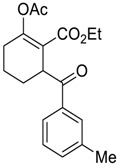 **124**	75
**3**	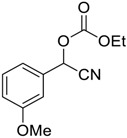 **8**	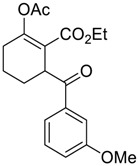 **125**	69
**4**	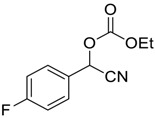 **5**	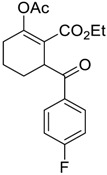 **126**	70
**5**	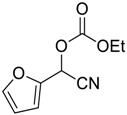 **14**	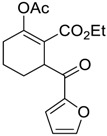 **127**	62
**6**	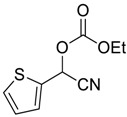 **13**	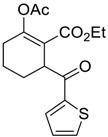 **128**	65
**7 ^c^**	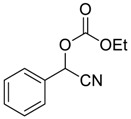 **1**	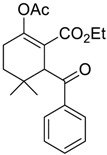 **129**	0
**8 ^d^**	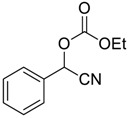 **1**	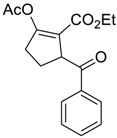 **130**	70

^a^ Reaction conditions: 1.0 mmol of ethyl carbonate of cyanohydrin, 2.2 mmol of (TMS)_2_NLi and 1.0 mmol of cycloalk-2-en-1-one were stirred in THF for 15 min. at −78 °C and 15 min. at 0 °C, followed by addition of 1.3 mmol of acetic anhydride and stirring at 0 °C for 3 h. ^b^ Isolated yields. ^c^ 4,4-dimethylcyclohex-2-en-1-one was used. ^d^ Cyclopent-2-en-1-one was used.

**Table 16:** Synthesis of aminofuranones via intramolecular Blaise reaction ^a^ [35].

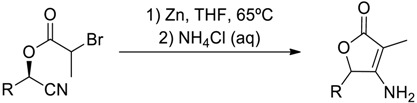				
Entry	Substrate	Product	Yield (%) ^b^	*er* ^c^
**1**	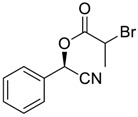	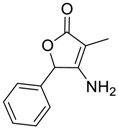 **219**	86	98.6:1.4
**2**	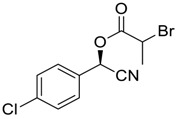	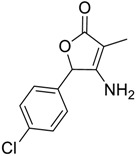 **220**	97	98.4:1.6
**3**	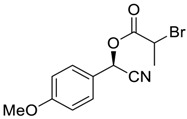	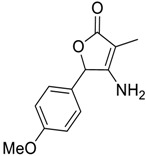 **221**	98	97.7:2.1
**4**	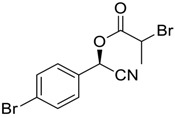	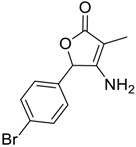 **222**	73	97.3:2.7
**5**	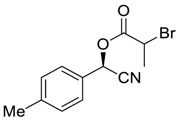	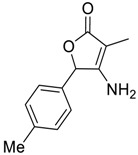 **223**	93	97.3:2.7
**6**	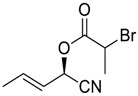	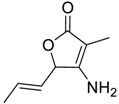 **224**	94	95.4:4.6
**7**	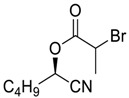	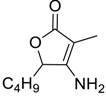 **225**	71	98.8:1.2

^a^ Reactions conditions: α-bromoacetylcyanohydrin (0.75 mmol) was added at 65 °C to a mixture of Zn (2.24 mmol) in THF. The mixture was cooled to −78 °C and NH_4_Cl (aq. sat.) was added. ^b^ Isolated yields. ^c^ Enantiomeric ratio determined by chiral GC or HPCL.

**Table 17:** Substrate scope for the cross-coupling of the *O*-(α-bromoacyl)cyanohydrin with boronic acids ^a^ [37].

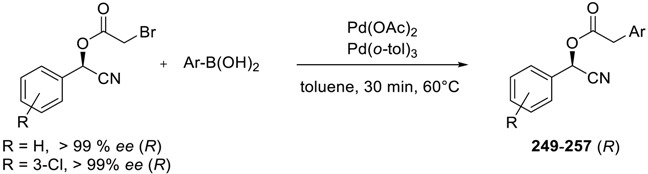					
Entry	Substrate	Boronic Acid	Product	Yield (%) ^b^	*ee* (%)
**1**	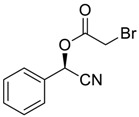	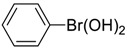	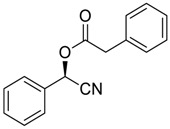 **249**	89	>99
**2**	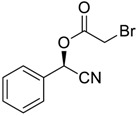	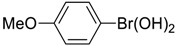	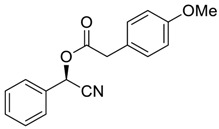 **250**	90	>99
**3**	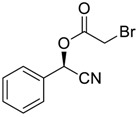	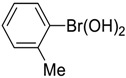	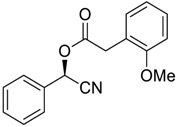 **251**	90	>99
**4**	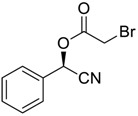	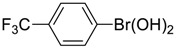	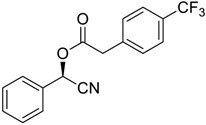 **252**	83	99
**5**	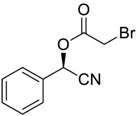	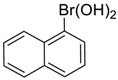	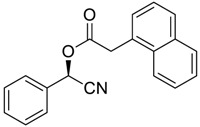 **253**	92	99
**6**	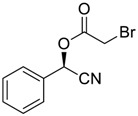	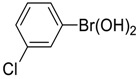	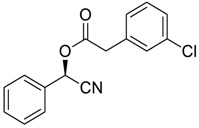 **254**	73	99
**7**	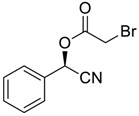	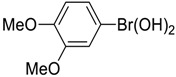	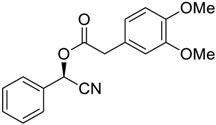 **255**	66	95
**8**	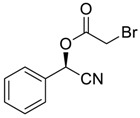	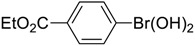	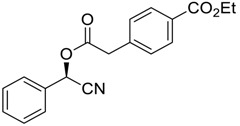 **256**	91	99
**9**	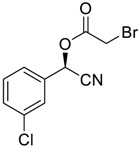	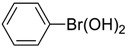	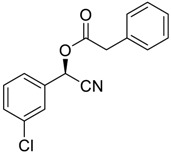 **257**	87	96

^a^ Reaction conditions: A solution of α-bromo acyl cyanohydrin (1 eq.) in toluene was added to a mixture of arylboronic acid (1.5 eq.), Pd(OAc)_2_ (3 mol %), (*o*-tol)_3_P (9 mol %)**,** KF (3 eq.) in toluene, the mixture was stirred at 60 °C. ^b^ Isolated yields.

**Table 18:** Titanium-mediated addition of EtMgBr to nitriles ^a^ [39].

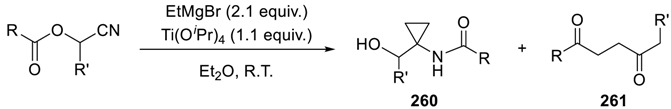					
Entry	Substract	Solvent	260/261 Ratio ^a^	260	261 (Yield %) ^b^
**1**	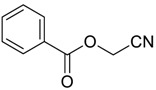	Et_2_O	14:86	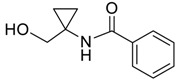 **260a**	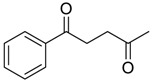 **261a** (69)
**2**	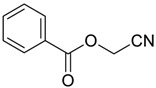	THF	37:63	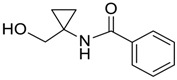 **260a**	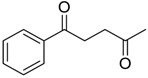 **261a** (45)
**3**	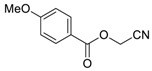	Et_2_O	13:87	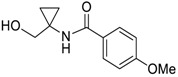 **260b**	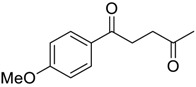 **261b** (65)
**4**	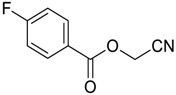	Et_2_O	7:93	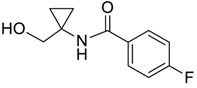 **260c**	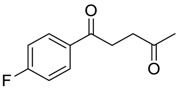 **261c** (60)
**5**	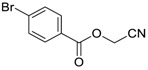	Et_2_O	20:80	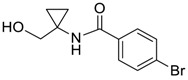 **260d**	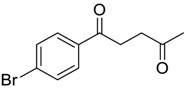 **261d** (60)
**6**	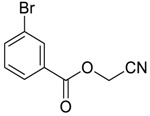	Et_2_O	60:40	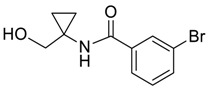 **260e**	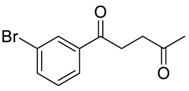 **261e** (74)
**7**	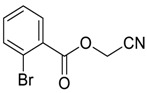	Et_2_O	7:93	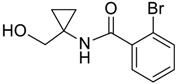 **260f**	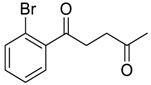 **261f** (62)
**8**	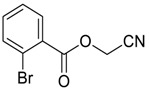	THF	22:78	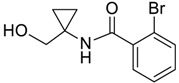 **260f** (40)	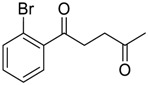 **261f** (31)
**9**	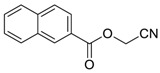	Et_2_O	7:93	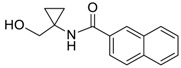 **260g**	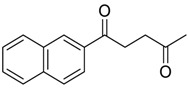 **261g** (62)
**10**	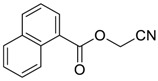	Et_2_O	22:78	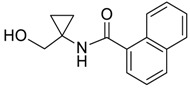 **260h**	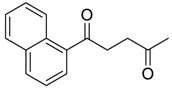 **261h** (66)
**11**	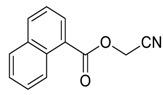	THF	59:41	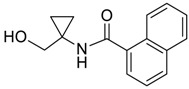 **260h**	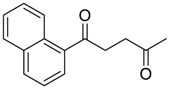 **261h** (32)
**12**	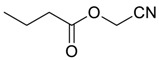	Et_2_O	7:93	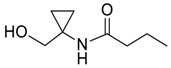 **260i**	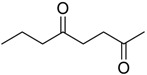 **261i** (50)
**13**	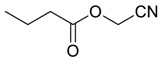	THF	13:87	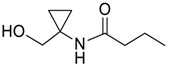 **260i**	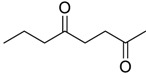 **261i** (48)
**14**	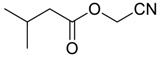	Et_2_O	13:87	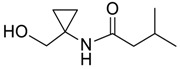 **260j**	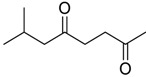 **261j** (50)
**15**	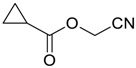	Et_2_O	8:92	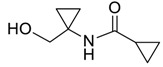 **260k**	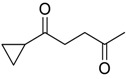 **261k** (55)
**16**	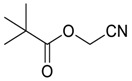	Et_2_O	20:80	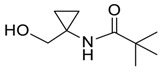 **260l**	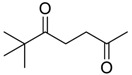 **261l** (56)
**17**	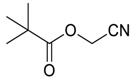	THF	90:10	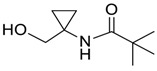 **260l**	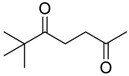 **261l** (60)
**18**	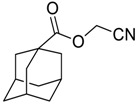	Et_2_O	30:70	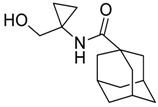 **260m**	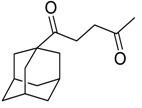 **261m** (58)
**19**	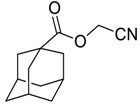	THF	81:19	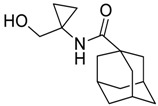 **260m**	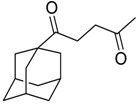 **261m** (69)
**20**	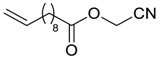	Et_2_O	12:88	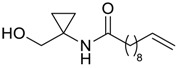 **260n**	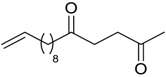 **261n** (62)
**21**	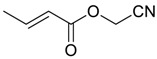	Et_2_O	3:97	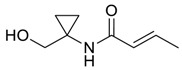 **260o**	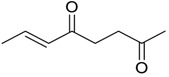 **261o** (42)
**22**	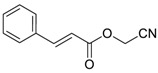	Et_2_O	8:92	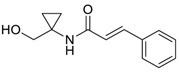 **260p**	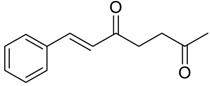 **261p** (45)
**23**	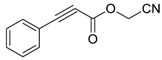	Et_2_O	22:78	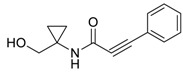 **260q**	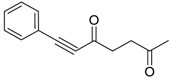 **261q** (42)
**24**	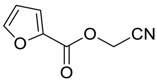	Et_2_O	12:88	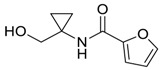 **260r**	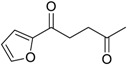 **216r** (50)
**25**	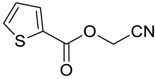	Et_2_O	19:81	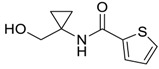 **260s**	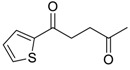 **261s** (65)
**26**	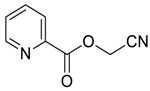	Et_2_O	[^c^]	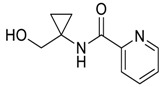 **260t**	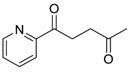 **261t** (0)
**27**	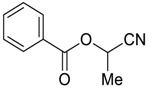	Et_2_O	15:85	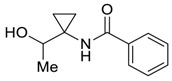 **260u**	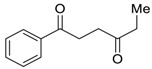 **261u** (56)
**28**	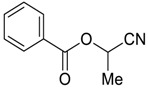	THF	28:72	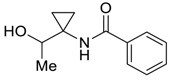 **260u**	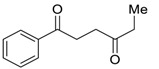 **261u** (52)
**29**	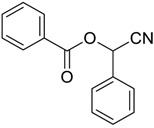	Et_2_O	22:78	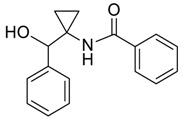 **260v**	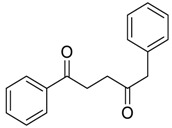 **261v** (52)
**30**	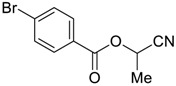	Et_2_O	12:88	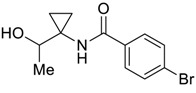 **260w**	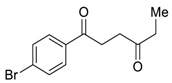 **261w** (50)
**31**	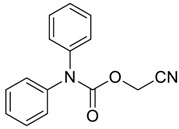	Et_2_O	54:46	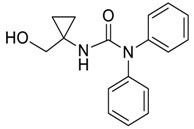 **260x** ^d^	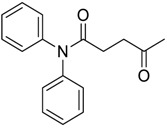 **261x** (34)
**32**	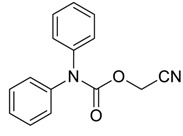	THF	57:43	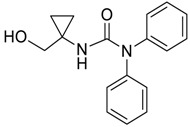 **260x** ^d^	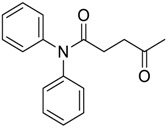 **261x** (27)
**33**	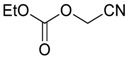	Et_2_O	100:0	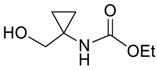 **260y** (31) + **262** (30) ^e^	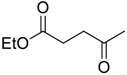 **261y**

^a^ Reaction conditions: To a solution of the nitrile (1 mmol) and Ti(O*^i^*Pr)_4_ (1.1 mmol) in Et_2_O or THF (10 mL) was added dropwise at 0 °C a solution of EtMgBr (*ca* 1 M in Et_2_O, 2.1 mmol) and stirred for 2 h. The **260**/**261** ratio was determined by ^1^H NMR spectroscopic analysis of the crude material. ^b^ Isolated yields after purification by chromatography. ^c^ Only the formation of isopropyl picolinate was observed. ^d^ Compound **260x** was contaminated with traces of an aromatic compound. ^e^ 6-oxa-4-azaspiro[2.4]heptan-5-one (**262**; 30%) was obtained together with **260y** (31%).

**Table 19:** Addition of EtMgBr to acyl cyanohydrins ^a^ [40].

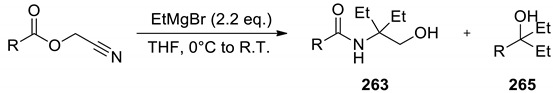				
Entry	Cyanohydrin	Product	263 Yield ^a^ (%)	Ratio ^b^ 263/265
**1**	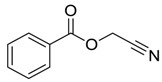	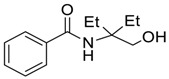 **263a**	65	83:17
**2 ^c^**	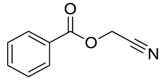	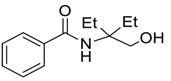 **263a**	14	26:74
**3**	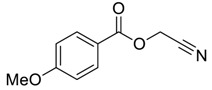	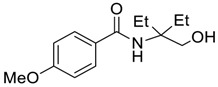 **263b**	73	84:16
**4**	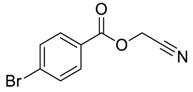	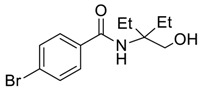 **263c**	58	71:29
**5**	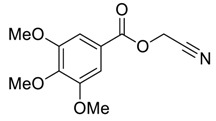	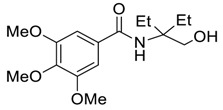 **263d**	68	84:16
**6**	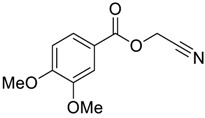	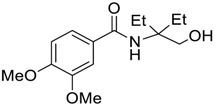 **263e**	65	87:13
**7**	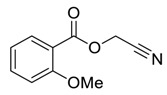	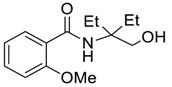 **263f**	^d^	0:100
**8**	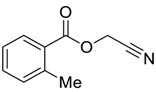	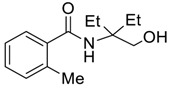 **263g**	72	96:4
**9**	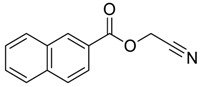	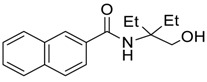 **263h**	59	71:29
**10**	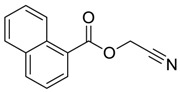	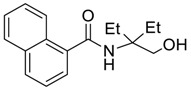 **263i**	74	96:4
**11 ^e^**	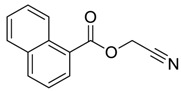	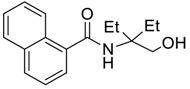 **263i**	77	>98:2

^a^ Reaction conditions: To a solution of the appropriate carboxylic acid (40 mmol) in CH_2_Cl_2_ (40 mL) cooled to 0 °C was added Et_3_N (80 mmol) and the mixture was stirred at room temperature for 10 min Chloroacetonitrile (60 mmol) was added. The mixture was stirred at room temperature. overnight. Isolated yields. ^b^ Ratio determined by ^1^H-NMR of the crude material. ^c^ Et_2_O used instead of THF. ^d^ Only 3-(2-methoxyphenyl)pentan-3-ol (**265f**) was obtained. ^e^ Reaction maintained at 0 °C for 30 min.

**Table 20:** Addition of Grignard reagents to acylcyanohydrin ^a^ [40].

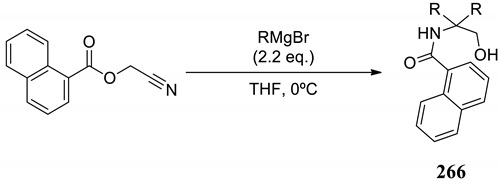			
Entry	Grignard Reagent	Product	Yield (%) ^a^
**1**	EtMgBr	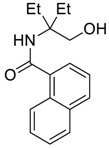 **266a**	77
**2**	MeMgBr	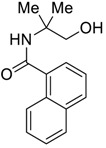 **266b**	73
**3**	*^n^*C_5_H_11_MgBr	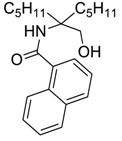 **266c**	68
**4**	Ph(CH_2_)_3_MgBr	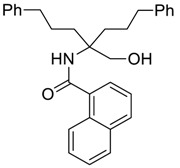 **266d**	79
**5**	PhMgBr	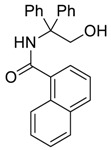 **266e**	70
**6**	4-MeO-C_6_H_4_MgBr	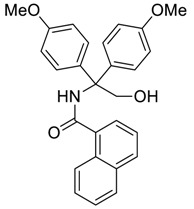 **266f**	82
**7**	H_2_C=CHMgBr	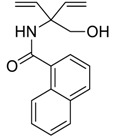 **266g**	71
**8**	H_2_C=CH(CH_2_)_2_MgBr	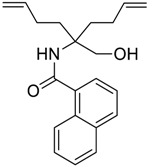 **266h**	72
**9**	H_2_C=CH(CH_2_)_3_MgBr	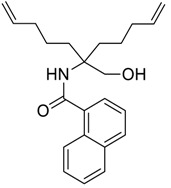 **266i**	60
**10**	H_2_C=CHCH_2_MgBr	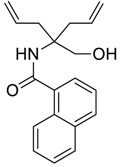 **266j**	26
**11**	H_2_C=CHCH_2_MgBr	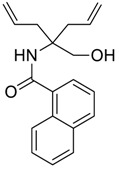 **266j**	57 ^b^

^a^ Reaction conditions: To a solution of cyanohydrin (5 mmol) in THF (25 mL) under N_2_ atmosphere and cooled to 0 °C was added dropwise the appropriate Grignard reagent (11 mmol). The reaction mixture was stirred for 30 min at 0 °C. Isolated yields. ^b^ Et_2_O used instead of THF.

**Table 21:** Two steps *versus* one step reaction to prepare 3-substituted-2-hydroxy-2-cyclopentenones [41].

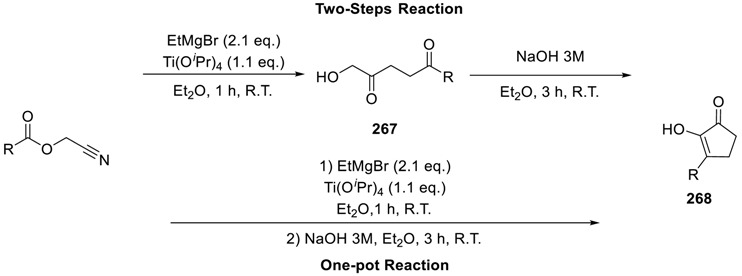				
Entry	Substrate	Product	Two-Step Yield	One-Pot ^a^ Yield
**1**	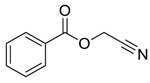	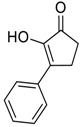 **268a**	62%	60% ^b^
**2**	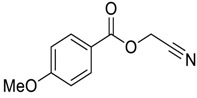	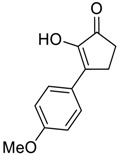 **268b**	49%	78%
**3**	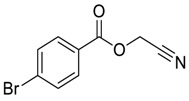	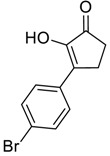 **268c**	52%	80%
**4**	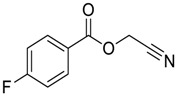	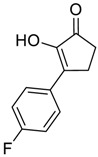 **268d**	46%	73%
**5**	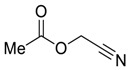	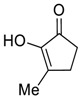 **268e**	-	72%
**6**	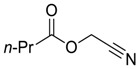	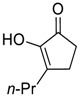 **268f**	-	68%
**7**	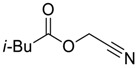	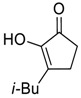 **268g**	-	63%
**8**	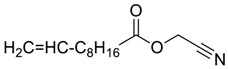	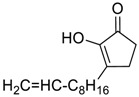 **268h**	-	69%
**9**	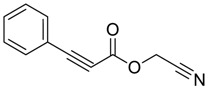	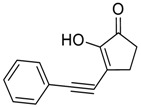 **268i**	-	21%

^a^ One-pot procedure: after the addition of EtMgBr to a solution of 1 mmol of cyanohydrin and Ti(O*^i^*Pr)_4_ in Et_2_O, the mixture was stirred for 1 h at room temperature.. A freshly prepared and degassed 3M aqueous NaOH solution was added, and vigorous stirring maintained for 3 h. ^b^ Reaction undertaken on 12 mmol of cyanohydrin.

**Table 22:** Scope of the rearrangement of *O*-aromatic acylated cyanohydrins ^a^ [43].

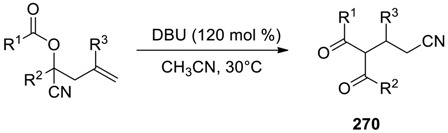					
Entry	Substrate	Product	Time (h)	Yield (%) ^b^	*dr* ^c^
**1**	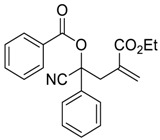	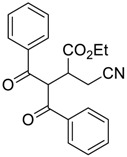 **270a**	43	67	-
**2**	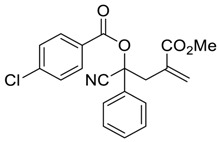	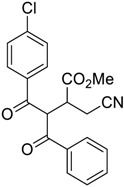 **270b**	24	65	1:1
**3**	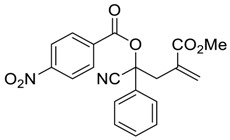	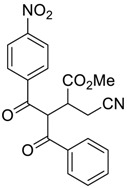 **270c**	1	32	1.1:1
**4**	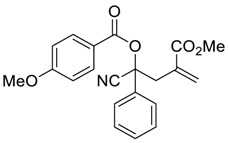	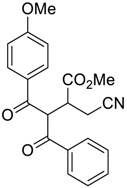 **270d**	53	31	1:1
**5**	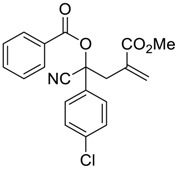	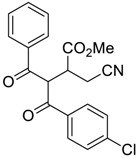 **270e**	6	83	1:1
**6**	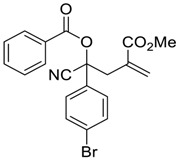	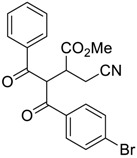 **270f**	7	77	1.2:1
**7**	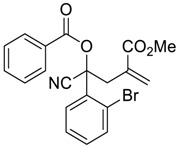	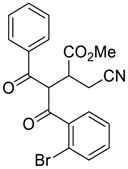 **270g**	48	38	1.1:1
**8**	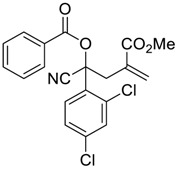	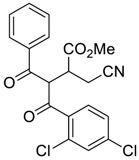 **270h**	16	60	1.1:1
**9**	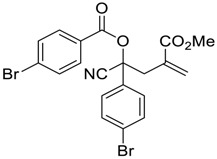	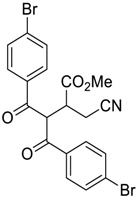 **270i**	6	56	-
**10**	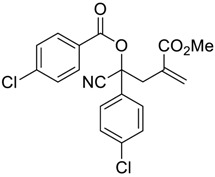	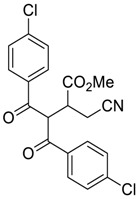 **270j**	3	53	-
**11**	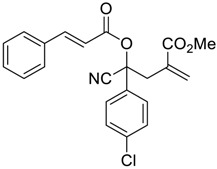	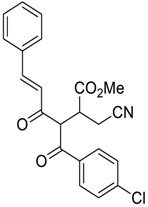 **270k**	1	72	1.4:1
**12**	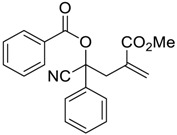	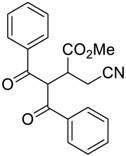 **270l**	24	38	-

^a^ Reactions were performed with the alkene (0.2 mmol) and DBU (120 mol %) in CH_3_CN. ^b^ Isolated yields. ^c^ diastereomeric ratio (*dr*) determined by ^1^H NMR analysis of crude products.

**Table 23:** Rearrangements of *O*-aliphatic acylated cyanohydrins ^a^ [43].

Entry	Substrate	Product	Time (h)	Yield (%) ^b^	*dr* ^c^
**1**	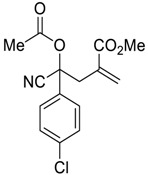	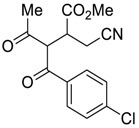 **270m**	1.5	78	1.1:1
**2**	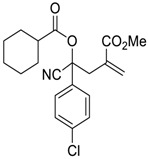	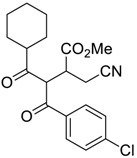 **270n**	4	54	1:1
**3 ^d^**	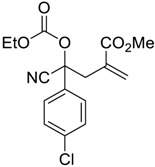	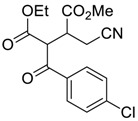 **270o**	5	-	-
**4**	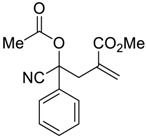	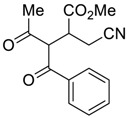 **270p**	2	77	1:1
**5**	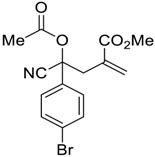	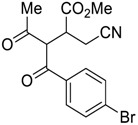 **270q**	2	63	1.2:1
**6**	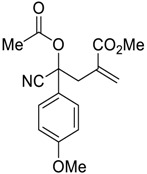	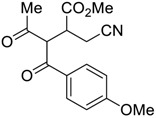 **270r**	6	72	1.1:1
**7**	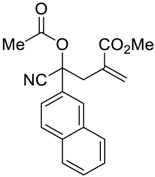	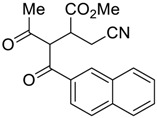 **270s**	3.5	69	1:1
**8**	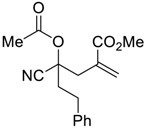	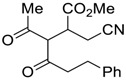 **270t**	6	20	1:1

^a^ Reactions were performed with alkene (0.2 mmol) and DBU (120 mol %) in CH_3_CN. ^b^ Isolated yields. ^c^ Determined by ^1^H NMR analysis of crude product. ^d^ No desired product was detected.

**Table 24:** Three components coupling reaction to form cyanohydrin derivatives ^a^ [45].

Entry	Alkyl Bromide	CO (atm)	Product	Yield (%) ^b^
**1 ^c^**	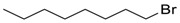	120	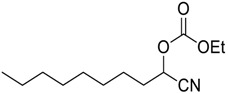 **273a**	79
**2**	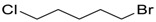	80	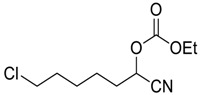 **273b**	60
**3**	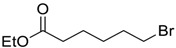	80	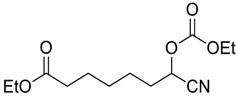 **273c**	83
**4**	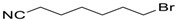	120	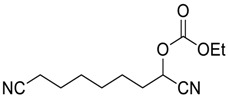 **273d**	76
**5**	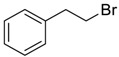	120	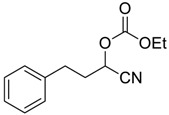 **273e**	61
**6**	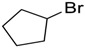	120	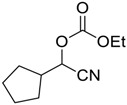 **273f**	61
**7**	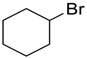	120	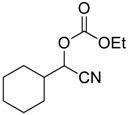 **273g**	74
**8**	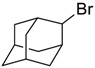	120	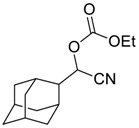 **273h**	73
**9**	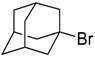	110	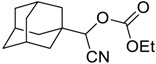 **273i**	82
**10**	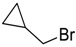	110	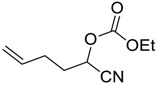 **273j**	45

^a^ Reaction conditions: alkyl bromide (0.05 mmol), CO 80 to 120 atm, EtOOCCN (1.6 eq.). ^b^ Isolated yields after flash chromatography on SiO_2._^c^ alkyl bromide (0.03 mmol).

## Data Availability

Not applicable.

## Abbreviations

Ac	Acetyl group
Acac	Acetylacetonate
AIBN	Azobisisobutyronitrile
Ar	Aryl group
BMIN	1-Butyl-3-methylimidazolium
bpy	2,2-Bipyridine
Bz	Benzyl group
DBU	1,8-Diazabicyclo[5.4.0]undec-7-ene
DCM	Dichloromethane
DMAP	4-Dimethylaminopyridine
DMF	Dimethylformamide
DMSO	Dimethyl sulfoxide
*dr*	diastereomeric ratio
DTAC	Dodecyltrimethylammonium chloride
DTMAC	4-[(*n*-dodecylthio)methyl]-7-(N,N-dimethylamino)-coumarin
EE	Ethoxyethyl acetal
*ee*	Enantiomeric excess
*er*	Enantiomeric ratio
GC	Gas chromatography
Gly	Glycine
HFIP	Hexafluoroisopropanol
HPLC	High-performance liquid chromatography
Me	Methyl
nd	not detected
NMA	N-Methylaniline
OEt	Ethoxy group
OMe	Methoxy group
SMA	Sulfa Michael Addition
Tf	Triflate
TFA	Trifluoroacetic acid
THF	Tetrahydrofuran
THP	Tetrahydropyran
TMS	Trimethylsilyl
TMSCN	Trimethylsilyl cyanide
TON	Turnover number
